# New and little-known species of the genus *Sphecodes* Latreille (Hymenoptera, Halictidae) from Southeast Asia

**DOI:** 10.3897/zookeys.937.51708

**Published:** 2020-06-01

**Authors:** Yulia V. Astafurova, Maxim Yu. Proshchalykin, Maximilian Schwarz

**Affiliations:** 1 Zoological Institute, Russian Academy of Sciences, Universitetskaya Nab., 1, Saint Petersburg 199034, Russia Zoological Institute, Russian Academy of Sciences Saint Petersburg Russia; 2 Federal Scientific Centre for East Asian Terrestrial Biodiversity, Far Eastern Branch of Russian Academy of Sciences, Vladivostok 690022, Russia Federal Scientific Centre for East Asian Terrestrial Biodiversity, Far Eastern Branch of Russian Academy of Sciences Vladivostok Russia; 3 A-4052, Ansfelden, Austria Unaffiliated Ansfelden Austria

**Keywords:** Anthophila, Apiformes, cleptoparasites, fauna, lectotype, taxonomy, distribution

## Abstract

The available information about the cleptoparasitic bees of the genus *Sphecodes* in Southeast Asia is summarized. Thirty-one species are currently known from this area. Four new species are described: *Sphecodes discoverlifei* Astafurova & Proshchalykin, **sp. nov.** (Laos), *S. engeli* Astafurova & Proshchalykin, **sp. nov.** (Laos, Vietnam), *S. ilyadadaria* Astafurova, **sp. nov.** (Indonesia), and *S. pseudoredivivus* Astafurova & Proshchalykin, **sp. nov.** (Laos). Nine species are newly recorded from South East Asia: *S. chaprensis* Blüthgen, 1927 (Laos), *S. howardi* Cockerell, 1922 (Malaysia, Myanmar, Thailand), *S. kershawi* Perkins, 1921 (Indonesia, Malaysia, Myanmar, Thailand), *S. laticeps* Meyer, 1920 (Thailand, Vietnam), *S. montanus* Smith, 1879 (Laos), *S. sauteri* Meyer, 1925 (Laos), *S. sikkimensis* Blüthgen, 1927 (Laos, Myanmar), *S. simlaensis* Blüthgen, 1924 (Laos), and *S. turneri* Cockerell, 1916 (Laos). Based on type specimens, new synonymies have been proposed for *Sphecodes kershawi* Perkins, 1921 = *S. javanensis* Blüthgen, 1927, **syn. nov.**; *S. simlaensis* Blüthgen, 1924 = *S. simlaellus* Blüthgen, 1927, **syn. nov.**; *S. laticeps* Meyer, 1920 = *S. biroi mariae* Cockerell, 1930, **syn. nov.** Lectotypes are designated for *Sphecodes biroi* Friese, 1909, *S. simlaellus* Blüthgen, 1927, and *S. laticeps* Meyer, 1920. The female of *Sphecodes sauteri* Meyer, 1925, and the male of *S. turneri* Cockerell, 1916 are described for the first time.

## Introduction

**Figure 1.**
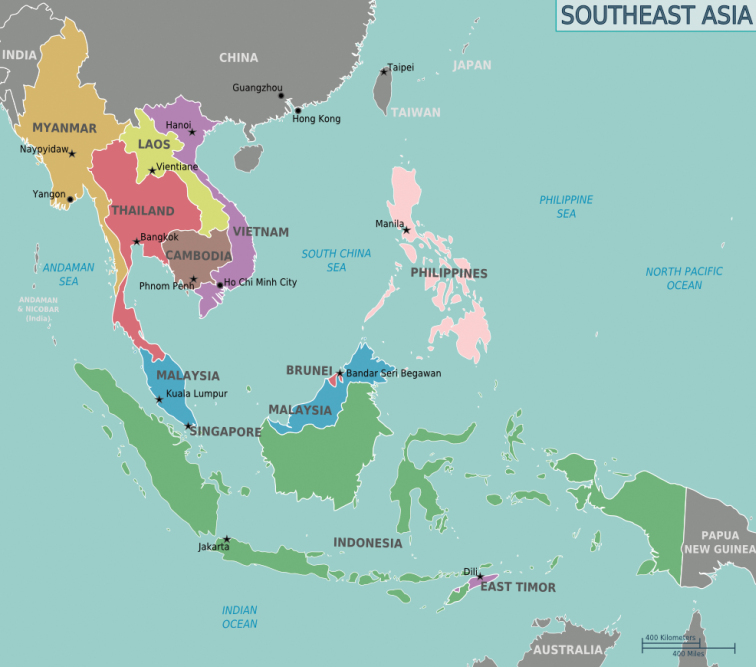
Map of Southeast Asia.

In recent years significant progress has been made towards a better knowledge of the species of *Sphecodes* Latreille from central and northern Asia (Astafurova and Proshchalykin 2014, 2015a, b, c, 2017a, b, 2018; Astafurova et al. 2015, 2018a, b, c, d, 2019). The purpose of this review is to improve our knowledge of the taxonomy and distribution of *Sphecodes* in Southeast Asia (Fig. 1) as an essential foundation for advancing biogeographical investigations in the Oriental Region.

Southeast Asia is composed of eleven countries of impressive diversity in habitats and landscapes: Brunei, Myanmar (Burma), Cambodia, East Timor, Indonesia, Laos, Malaysia, the Philippines, Singapore, Thailand and Vietnam (Fig. 1). This region has one of the highest concentrations of endemic species on Earth (Myers et al. 2000; Sloan et al. 2014; Sing et al. 2016) but knowledge of its bee fauna other than the relatively well-studied highly eusocial hive bees (Apini and Meliponini) remains very limited and inaccessible (Ascher et al. 2016). Currently 975 Southeast Asian species of Halictidae from fifteen genera are recognized as valid (Ascher and Pickering 2019), but the taxonomy and distribution of these species requires much additional study. We begin here with reference to the genus *Sphecodes* Latreille.

The first information on the genus *Sphecodes* from Southeast Asia was published by Bingham (1897), who record *S. apicatus* Smith [= *S. gibbus* (Linnaeus)] from the Pegu Hills, Burma (= Myanmar). But it is obvious that this is a misidentification and the record actually refers to another large *Sphecodes* species. *Sphecodes brunneipes* Friese, 1914 was the first species of the genus *Sphecodes* described from Southeast Asia (Indonesia) and since then eleven additional species have been described from this area by Cockerell 1915, 1919, 1930 (four species), Blüthgen 1924, 1927 (four species), and Meyer 1925 (three species), with only seven of them still valid (current data). Until now there has been no review published on Southeast Asian *Sphecodes* and all information on the distribution of the 17 known species was only available on the Discover Life website (Ascher and Pickering 2019).

In the present paper, based on a comprehensive study of specimens (including primary types) held in various collections, we report additional records of 21 species, with four species described as new and nine species recorded from Southeast Asia for the first time, resulting in a total number of 31 *Sphecodes* species known from this region (Table 1). The genus *Sphecodes* has not yet been documented from Brunei, Cambodia, and East Timor although it is probable that this cosmopolitan genus is present in these countries and it is only a matter of time before the fauna is sampled and recorded.

In addition, we describe the female of *Sphecodes sauteri* Meyer, 1925, and the male of *S. turneri* Cockerell, 1916 for the first time, propose new synonymies for three specific names (*S. kershawi* Perkins, 1921 = *S. javanensis* Blüthgen, 1927, syn. nov.; *S. laticeps* Meyer, 1920 = *S. biroi mariae* Cockerell, 1930, syn. nov.; *S. simlaensis* Blüthgen, 1924 = *S. simlaellus* Blüthgen, 1927, syn. nov.), and designate lectotypes for *Sphecodes biroi* Friese, 1909, *S. laticeps* Meyer, 1920, and *S. simlaellus* Blüthgen, 1927 in order to clarify the status and diagnosis of type specimens.

**Table 1.** Checklist of the *Sphecodes* species of Southeast Asia including distribution by countries.

Species		Southeast Asia							
		Indonesia	Laos	Malaysia	Myanmar	Philippines	Singapore	Thailand	Vietnam
No. collecting sites		7	4	8	3	3	–	3	4
No. bees examined		11	16	11	3	6	–	22	8
1	*S. bakeri* Cockerell	●				○●			
2	*S. binghami* Blüthgen			●	○				
3	*S. biroi* Friese	○●		●		○		●	
4	*S. bischoffi* Blüthgen	○			○				
5	*S. brunneipes* Friese	○●							
6	*S. chaprensis* Blüthgen		●						
7	*S. discoverlifei* Astafurova & Proshchalykin, sp. nov.		●						
8	*S. distinctus* Meyer							●	○
9	*S. duplex* Blüthgen	○●		●			○		
10	*S. engeli* Astafurova & Proshchalykin, sp. nov.		●						●
11	*S. fumipennis* Smith		●		○				
12	*S. howardi* Cockerell			●	●			●	
13	*S. ilyadadaria* Astafurova, sp. nov.	●							
14	*S. insularis* Smith	○							
15	*S. javanicus* Friese	○							
16	*S. kershawi* Perkins	○●		●	●			●	
17	*S. laticeps* Meyer							○	●
18	*S. luzonicus* Blüthgen					○			
19	*S. malayensis* Blüthgen			○					
20	*S. montanus* Smith		●						
21	*S. pseudoredivivus* Astafurova & Proshchalykin, sp. nov.		●						
22	*S. redivivus* Blüthgen	○							
23	*S. rotundiceps* Cockerell					○			
24	*S. samarensis* Blüthgen	●		●		○			
25	*S. sauteri* Meyer		●						
26	*S. sibuyanensis* Cockerell					○			
27	*S. sikkimensis* Blüthgen		●		●				
28	*S. simlaensis* Blüthgen		●						
29	*S. tertius* Blüthgen				○				
30	*S. tristellus* Cockerell					○			
31	*S. turneri* Cockerell		●						
	**Total**:	11	10	7	7	7	1	5	3

Circle – published records (Cockerell 1919, 1925, 1930, Meyer 1920, 1925, Blüthgen 1924, 1927, Ascher and Pickering 2019); black circle – current data. Genus *Sphecodes* are not known in Brunei, Cambodia, and East Timor.

## Materials and methods

The results presented in this paper are based on 77 specimens collected in Southeast Asia and currently housed in the Natural History Museum (London, UK, **NHMUK**); National Museum of Natural History, Smithsonian Institution, Washington, DC, USA (**USNM**), the Zoological Institute, Russian Academy of Sciences (St. Petersburg, Russia, **ZISP**); Museum für Naturkunde der Humboldt Universität zu Berlin, Germany (**ZMHB**), Senckenberg Deutsches Entomologisches Institut, Müncheberg, Germany (**SDEI**), Zoologische Staatssammlung, München, Germany (**ZSM**), Hungarian Natural History Museum, Budapest, Hungary (**HNHM**), Oberösterreichisches Landesmuseum, Biologiezentrum, Linz, Austria (**OLBL**) and the personal collection of Maximilian Schwarz (Ansfelden, Austria, **PCMS**).

Morphological terminology follows that of Engel (2001) and Michener (2007). The ventral surface of some flagellomeres bear a distinctive patch of sensilla trichodea A (sensu Årgent and Svensson 1982), which we refer to as ‘tyloids’, easily observable under the microscope. Abbreviations F, T, and S are used for flagellomere, metasomaltergum and metasomal sternum respectively. The density of integumental punctures is described using the following formula: puncture diameter (in μm) / ratio of distance between punctures to average puncture diameter, e.g., 15–20 μm / 0.5–1.5. Integumental sculpture other than distinctive surface punctation is described following Harris (1979): areolate – coarse, contiguous punctures; reticulate – superficially net-like or network of raised lines; rugose – irregular, nonparallel, wrinkled raised lines (rugae); rugulose – minutely rugose; strigate – narrow, transverse or longitudinal streaks (strigae), variety of parallel lineations; tessellate – regular network of shallow grooves with flat interspaces.

Specimens were studied with a Leica M205A stereomicroscope and photographs taken with a combination of stereomicroscope (Olympus SZX10) and digital camera (Olympus OM-D and Canon EOS70D). Final images are stacked composites using Helicon Focus 6. All images were post-processed for contrast and brightness using Adobe Photoshop.

New distributional records are noted with an asterisk (*).

## Taxonomy

### List of species

#### Sphecodes bakeri

Taxon classificationAnimaliaHymenopteraHalictidae

Cockerell, 1915

7E599CAF-9378-58ED-8651-F73F2C3BC319

Figures 3 , 5

Sphecodes bakeri Cockerell, 1915: 489, ♀ (holotype: ♀, Philippines, Dapitan, Mindanao, Baker leg.; USNM, http://n2t.net/ark:/65665/34c597e0b-f31f-4bd8-82fb-e3e6a1379222).

##### Diagnosis.

Structurally and sculpturally this species is very similar to the male of *Sphecodes samarensis* Blüthgen, 1927 and the female of *S. duplex* Blüthgen, 1927, but from the first species it differs by weakly developed antennal tyloids (versus tyloids covering large part of ventral flagellar surface in *S. samarensis*) and from the second species by the ocello-ocular area (Fig. 5) having entirely confluent punctures (versus the ocello-ocular area with narrow shiny interspaces in *S. duplex*, Fig. 4).

##### Descriptive notes.

Wings with weak yellow-brownish darkening; hind wing with angle between basal (M) and cubital (Cu) veins ca. 70°, costal margin with eight hamuli. Lateral preoccipital carina present. **Female.** Total body length 6–8 mm. Head strongly transverse in front view, ca. 1.3 times as wide as long (Fig. 3); vertex weakly elevated with distance from top of head to upper margin of lateral ocellus approximately a lateral ocellar diameter as seen in frontal view; labrum trapezoidal, 0.6 times as long as basal width; face and ocello-ocular area with confluent punctures; paraocular and supraclypeal areas with adpressed white pubescence obscuring integument, gena with sparser pubescence. Mesoscutum and mesoscutellum areolate-punctate (30–50 μm); propodeal triangle (metapostnotum) and lateral parts of propodeum with longitudinal wrinkles and smooth shiny interspaces between them; mesepisternum reticulate-rugose. Metasomal terga almost impunctate, sometimes with a few fine punctures, red; pygidial plate widely rounded apically, 1.2–1.4 times as wide as metabasitarsus. **Male.** Total body length 5.5–6.5 mm. Head transverse, ca. 1.15 times as wide as long; vertex weakly elevated with distance from top of head to upper margin of lateral ocellus approximately a lateral ocellar diameter as seen in frontal view; tyloids weakly developed, narrowly linear as seen in lateral view; F2 1.6 times as long as wide; F3 = F4, 1.2–1.3 times as long as wide; face (below andabove the antennal sockets) with adpressed white pubescence obscuring integument. Mesoscutum and mesoscutellum areolate-punctate; propodeum and mesepisternum as in the female. Metasomal terga finely punctate (15–20 μm / 1–3); marginal zonesimpunctate; T1–T3 red; gonocoxite dorsally without impression; gonostylus short, apically broadened.

**Figures 2–7.**
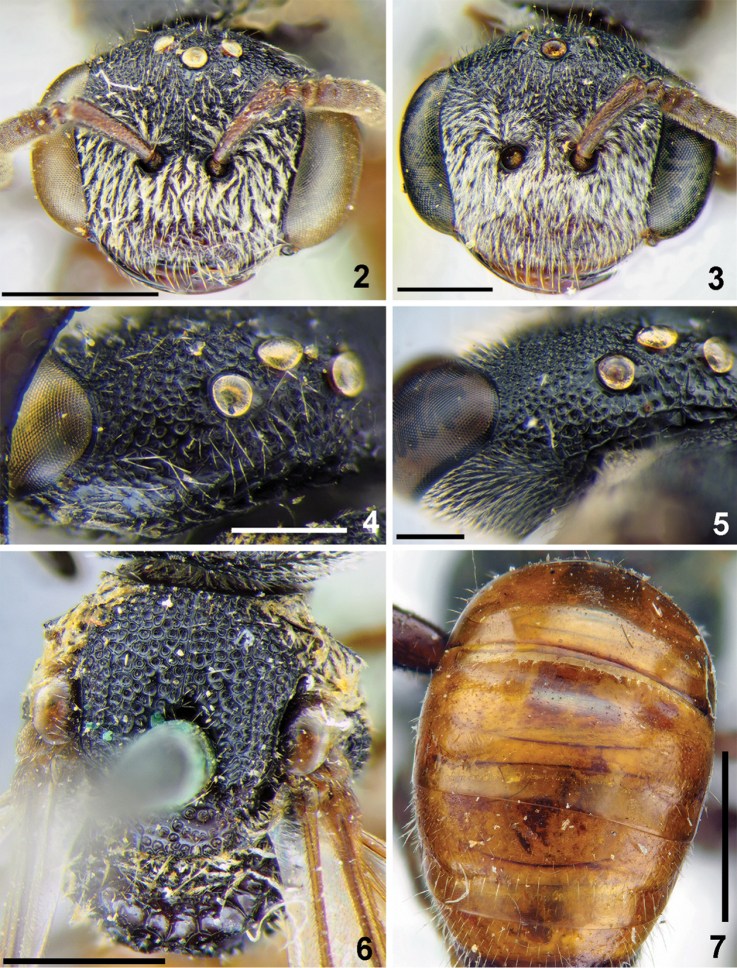
*Sphecodes duplex* Blüthgen (2, 4, 6, 7), *S. bakeri* Cockerell (3, 5), females **2, 3** head, frontal view **4, 5** vertex, dorso-lateral view **6** mesosoma, dorsal view **7** T1–T3, dorsal view. Scale bars: 1.0 mm (**2, 3, 6, 7**), 0.5 mm (**4, 5**).

##### Material examined.

Indonesia: 1 ♀, NE Sulawesi, 47 km WSW Kotamobagu, Dumoga-Bone N. Pk., Toraut, 211 m, VII.1985, G.R. Else (NHMUK 013380350); Philippines: 1 ♂, Dapitan, Mindano, Baker leg. [*Sphec. bakeri* Ckll., Blüthgen det.] (ZMHB); 1 ♀, Kolambugan, Mindanao, Baker leg. [*Sphec. bakeri* Ckll., Blüthgen det.] (ZMHB).

##### Published records.

Cockerell 1915: 489 (Philippines); Blüthgen 1927: 74 (Philippines); Ascher and Pickering 2019 (Philippines).

##### Distribution.

*Indonesia, Philippines.

#### Sphecodes binghami

Taxon classificationAnimaliaHymenopteraHalictidae

Blüthgen, 1924

8A4F707F-F45F-5B96-95FA-946E6340B50F

Figures 8–11

Sphecodes binghami Blüthgen, 1924: 497, ♀ (holotype: ♀, Myanmar, Pegu Hill, 3.89, coll. Bingham; NHMUK 010576231; examined).

##### Diagnosis.

The female of this species resembles *Sphecodes takaensis* Blüthgen, 1927 owing to a similar structure, sculpture and coloration of the body, but it differs from this species by the square F3 (as long as wide), the entirely areolate vertex and the wider pygidial plate which is as wide as metabasitarsus (in *S. takaensis* F3 0.7–0.8 times as long as wide; vertex with small shiny impunctate spots near ocelli; pygidial plate narrower than metabasitarsus).

##### Descriptive notes.

**Figures 8–11.**
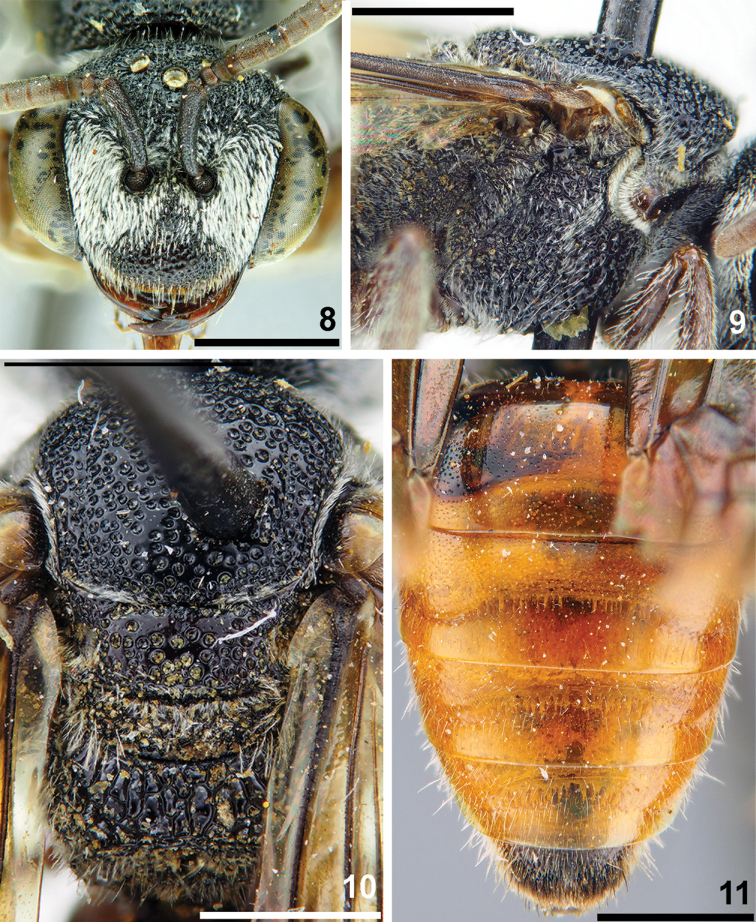
*Sphecodes binghami* Blüthgen, female **8** head, frontal view **9, 10** mesosoma, lateral view (**9**), dorsal view (**10**) **11** metasoma, dorsal view. Scale bars: 1.0 mm.

Wings with brownish darkening; hind wing with the angle between basal (M) and cubital (Cu) veins ca. 80°, costal margin with eight or nine hamuli. Lateral preoccipital carina present. **Female.** Total body length 8–9 mm. Head (Fig. 8) transverse, ca. 1.25 times as wide as long; vertex weakly elevated with distance from top of head to upper margin of lateral ocellus approximately a lateral ocellar diameter as seen in frontal view; labrum trapezoidal, 0.45 times as long as basal width; face and vertex areolate-punctate; paraocular (below and above the antennal sockets), supraclypeal areas and gena with adpressed white pubescence obscuring integument. Mesoscutum and mesoscutellum (Fig. 10) densely punctate (40–75 μm), medially with punctures separated by at most 1–2 puncture diameters, becoming confluent peripherally; propodeal triangle (metapostnotum) and mesepisternum (Fig. 9) reticulate-rugose. Metasoma red (Fig. 11); T1 on disc and marginal zone finely and densely punctate (10–15 μm / 0.5–3), remaining terga more coarsely punctate (10–25 μm) with impunctate and smooth marginal zones; pygidial plate dull, widely rounded apically, as wide as metabasitarsus. **Male** unknown.

##### Material examined.

Malaysia: 1 ♀, Malaya, S. Batu Feringgi, Panang, 4.VIII.1955, H.T. Pagden (NHMUK 013380344).

##### Published records.

Blüthgen 1924: 497 (Myanmar).

##### Distribution.

*Malaysia, Myanmar.

#### Sphecodes biroi

Taxon classificationAnimaliaHymenopteraHalictidae

Friese, 1909

3BBBC0D8-4B86-5911-BABC-9D23438AF5B4

Figures 12–16 , 17–21

Sphecodes biroi Friese, 1909 (nec Meyer, 1925): 181, ♀, ♂ (lectotype (**designated here**): ♂, N. Guinea, Biro 1899 // Sattelberg, Huon-Golf. // Sphecodes biroi Fiese det., 1908, ♂ // Type // Lectotypus, Sphecodes biroi Friese, 1909, design. Astafurova et Proshchalykin, 2020 <red label>), Fig. 16; Paralectotype: ♀, the same label (Fig. 16); HNHM, examined.
Sphecodes transversus Cockerell, 1919: 556, ♀ (holotype: ♀, Philippines, Luzon, Mt Makiling, Baker leg.; USNM, http://n2t.net/ark:/65665/372106fa4-0a44-4b85-812d-254423957856). Synonymized by Blüthgen 1927: 79.
Sphecodes latifrons Cockerell, 1919: 556, ♂ (holotype: ♂, Philippines, Luzon, Baguio, Benguet, Baker leg.; USNM, http://n2t.net/ark:/65665/3ed6e3af8-0ca2-4dac-a230-436c053d6475). Synonymized by Blüthgen 1927: 79.
Sphecodes abnormis Perkins, 1921: 10–11, ♂ (holotype: ♂, “East Indies”; ?). Synonymized by Blüthgen 1927: 79.
Sphecodes amboinensis Meyer, 1925: 11, ♂ (syntypes: 4 ♂♂, Indonesia, Amboina, 1998, Biro leg.; HNHM). Synonymized by Blüthgen 1927: 79.
Sphecodes bischoffi Meyer, 1925 (nom. praeocc., nec S. bischoffi Blüthgen, 1924): 11, ♂ (holotype: ♂, Indonesia, Java, Buitenzorg; ZMHB). Synonymized by Blüthgen 1927: 79.

##### Diagnosis.

This species resembles *Sphecodes laticeps* Meyer, 1920 in having a similar structure, sculpture and coloration of the body, including the shape of the male genitalia. However, the male differs in the size of tyloids which usually cover the entire ventral flagellar surface or sometimes with a small glabrous spot on basal flagellomeres (versus antennae with well-developed medial glabrous spot on ventral surface of flagellomeres in *S. laticeps*); females are challenging to distinguish, but *S. laticeps* has T2 usually more distinctly punctate.

##### Descriptive notes.

**Figures 12–16.**
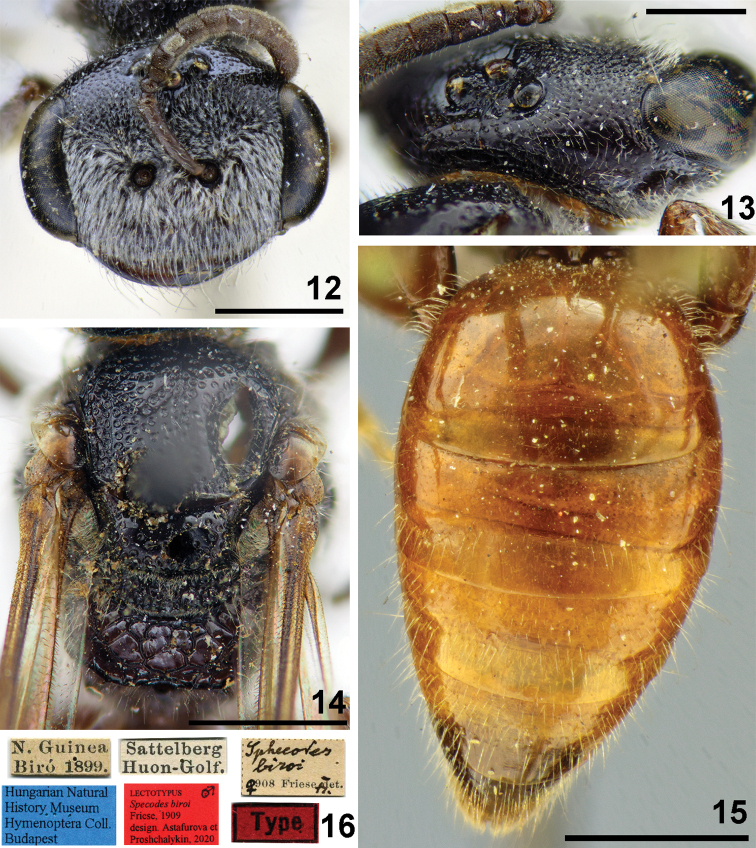
*Sphecodes biroi* Friese, female, paralectotype **12** head, frontal view **13** vertex, dorso-lateral view **14** mesosoma, dorsal view **15** metasoma, dorsal view **16** labels. Scale bars: 1.0 mm (**12, 14, 15**), 0.5 mm (**13**).

**Figures 17–21.**
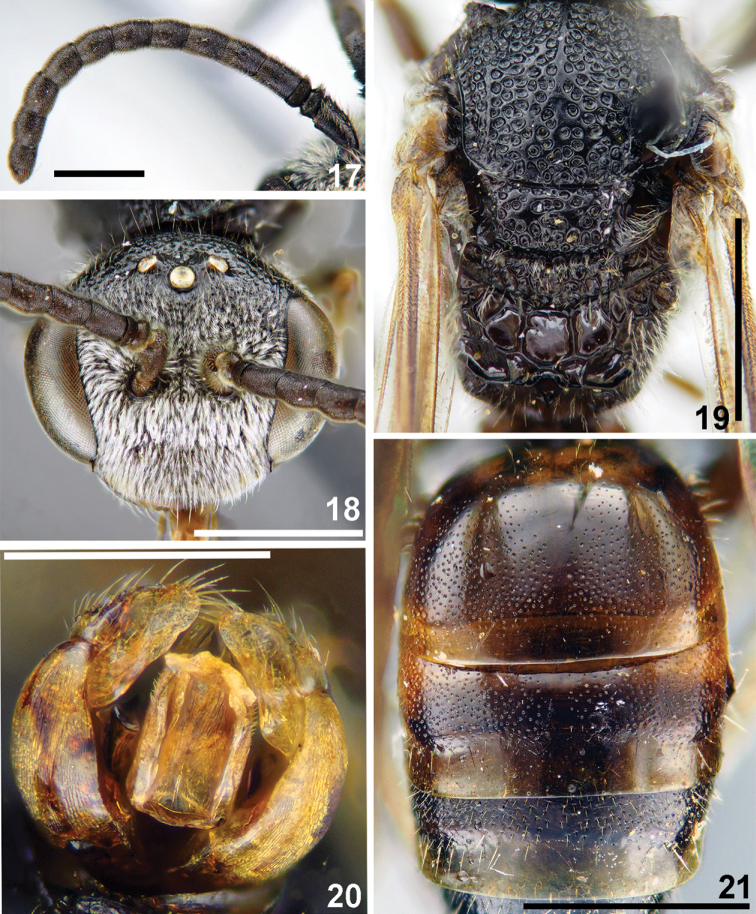
*Sphecodes biroi* Friese, male **17** antennae, lateral view **18** head, frontal view **19** mesosoma, dorsal view **20** genitalia, dorsal view **21** T1-T3, dorsal view. Scale bars: 1.0 mm (**17–19, 21**), 0.5 mm (**20**).

Wings with weak yellow-brownish darkening; hind wing with the angle between basal (M) and cubital (Cu) veins ca. 80°, costal margin with seven hamuli. Lateral preoccipital carina present. **Female.** Total body length 6.5–7.5 mm. Head (Fig. 12) strongly transverse, ca. 1.3 times as wide as long; vertex elevated with distance from top of head to upper margin of lateral ocellus 1–1.5 times lateral ocellar diameter as seen in frontal view; labrum trapezoidal, 0.6 times as long as basal width; vertex (Fig. 13) with shiny interspaces between shallow punctures (approximately a puncture diameter); paraocular (below the antennal sockets) and supraclypeal areas with adpressed white pubescence obscuring integument, clypeus and gena with sparser pubescence. Mesoscutum and mesoscutellum (Fig. 14) mostly with confluent punctures (30–40 μm) and medially with a few shiny interspaces at most 1–2 puncture diameters; propodeal triangle (metapostnotum) roughly reticulate-rugose (sculpture forming 1–2 rows of large deep cells); mesepisternum reticulate-rugose. Metasomal T1 impunctate or with a few tiny punctures (Fig. 15), T2 medially impunctate or with tiny and sparse punctures, but coarser and denser on lateral parts (10–15 μm / 2–4); marginal zones impunctate; pygidial plate as wide as metabasitarsus; T1 and T2 red, T3 and T4 red or dark. **Male.** Total body length 6–7 mm. Head transverse (Fig. 18), ca. 1.2 times as wide as long; vertex elevated with distance from top of head to upper margin of lateral ocellus 1–1.5 times lateral ocellar diameter as seen in frontal view; antennae attain posterior margin of mesoscutum, F2 1.4–1.5 times as long as wide; tyloids well developed, covering entire ventral and lateral flagellar surfaces (Fig. 17). Mesoscutum and mesoscutellum (Fig. 19) mostly areolate with a few shiny interspaces at most a puncture diameter; propodeal triangle roughly reticulate-rugose (sculpture forming one or two rows of large deep cells); lateral parts of propodeum rugose with large smooth shiny interspaces. Metasomal terga (Fig. 21) with fine and relatively dense punctures (10–15 μm / 1–3), punctures more visible in specimens with darker terga; T1–T3 red or brownish; gonocoxite dorsally without impression; gonostylus as on Fig. 20.

##### Material examined.

Malaysia: 1 ♂, Malaya, Kuala Lumpur, 17.XI.1929, H.T. Pagden (NHMUK 013380438); 1 ♀, idem, Batu Road, 19.VII.1936, H.T. Pagden (NHMUK 013380348); 1 ♂, Perak Gerik env., 26–28.II.2000, K. Denes jun. (OLBL/PCMS); 3 ♂♂, Perak Bakit Larut, 23–25.II.2000, K. Denes jun. (OLBL/PCMS); Indonesia: 1 ♀, NE Sulawesi, 47 km WSW Kotamobagu, Dumoga-Bone N. Pk., Toraut, 211 m, VII.1985, G.R. Else (NHMUK 013380352); 1 ♂, North Sumatra,Brastagi, 76 km S Medan, 3.III–1.IV.1996, S. Becvar (OLBL/PCMS); Thailand: 2 ♀♀, 2 ♂♂, Nan, MaeCharim NPEingang, 18°36'N, 100°58'E, 206 m, 13–22.V.2012, E. & J. Holzschuh (OLBL/PCMS); 1 ♂, Phuket Island, Main Harn, 1–5.II.2018, J. Halada (OLBL/PCMS); 1 ♂, Chumphon prov., 27.III–14.IV.1996, 9°48'N, 98°47'E, P. Prudek (OLBL/PCMS); Sri Lanka: ♂, Sri Lanka, Gal. Dist., Kanneliya Section, Sinharaja, 2–5.X.1980, K. Krombein et al. leg. (USNM) (S. Sakagami det as. ”*Sphecodes lankensis*” – unpublished manuscript name).

##### Published records.

Cockerell 1919: 556 (Philippines, as *S. transversus* and *S. latifrons*); Meyer 1920: 230 (Philippines, as *S. insularis*); 1925: 11 (Indonesia, as *S. bischoffi*); Perkins, 1921: 10 (East India, as *S. abnormis*); Ascher and Pickering 2019 (Indonesia, Philippines).

##### Distribution.

Indonesia, *Malaysia, Philippines, *Thailand, India, Sri Lanka, New Guinea.

##### Remarks.

Records of this species in Thailand (Ascher and Pickering 2019) refer to *Sphecodes biroi mariae* Cockerell, 1930 = *S. laticeps* Meyer, 1920 (see below).

#### Sphecodes brunneipes

Taxon classificationAnimaliaHymenopteraHalictidae

Friese, 1914

4C25FE7C-6803-5425-9564-469EDC7CE95E

Figures 22–27

Sphecodes brunneipes Friese, 1914: 14, ♀ (holotype: ♀, Indonesia, Java, Buitzotg. Schmiedek. leg., Coll. Friese; ZMHB; examined, Fig. 27).

##### Diagnosis.

Unlike other species with simple mandibles in the female, this species has a preoccipital carina and a weakly curved basal vein in hind wing.

##### Descriptive notes.

**Figures 22–27.**
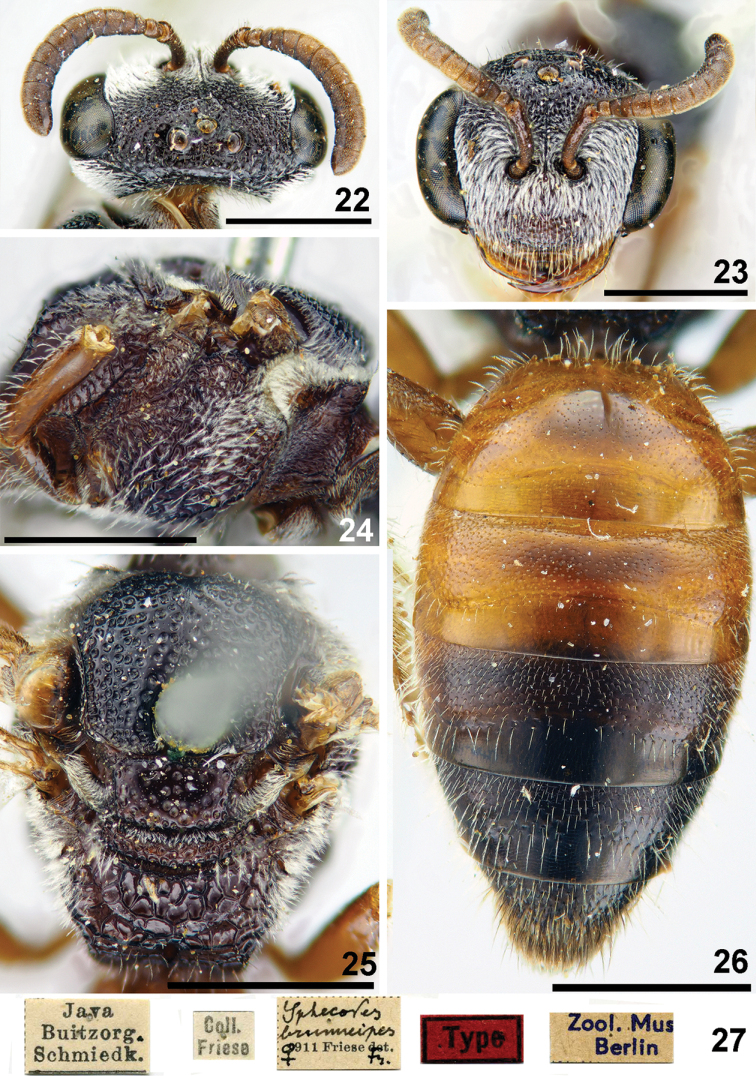
*Sphecodes brunneipes* Friese, female, holotype **22, 23** head, dorsal view (**22**), frontal view (**23**) **24, 25** mesosoma, lateral view (**24**), dorsal view (**25**) **26** metasoma, dorsal view **27** labels. Scale bars: 1.0 mm.

Wings with brownish darkening; hind wing with the angle between basal (M) and cubital (Cu) veins ca. 70°, costal margin with seven hamuli. Lateral and dorsal preoccipital carina present. **Female.** Total body length 6–7 mm. Head (Fig. 23) strongly transverse, ca. 1.25 times as wide as long; vertex weakly elevated with distance from top of head to upper margin of lateral ocellus approximately a lateral ocellar diameter as seen in frontal view; F1 and F2 strongly transverse, 0.5 times as long as wide; F3 0.8 times as long as wide; face with confluent punctures, ocello-ocular area with dense punctures separated by at most a puncture diameter (Fig. 22); paraocular and supraclypeal areas with adpressed white pubescence obscuring integument. Gena with dense pubescence. Mesoscutum and mesoscutellum (Fig. 25) coarsely and densely punctate (25–50 μm), the punctures separated by at most two puncture diameters; mesepisternum reticulate-rugose (Fig. 24); propodeal triangle (metapostnotum) coarsely reticulate-rugose, lateral parts of propodeum with fine wrinkles (strigose). Metasomal terga definitely punctate (Fig. 26), finely on T1 (ca. 10 μm) and more coarsely on the remaining terga (10–25 μm); marginal zones impunctate; T1 and T2 red, coloration of T3 and T4 variable; pygidial plate dull, widely rounded apically, 1.4 times as wide as metabasitarsus. **Male** unknown.

##### Published records.

Friese 1914: 14 (Indonesia); Ascher and Pickering 2019 (Indonesia).

##### Material examined.

Indonesia: 2 ♀♀, Lombok, near Senggigi, 18.V.2012, M. Mokrousov (ZISP).

##### Distribution.

Indonesia.

#### Sphecodes chaprensis

Taxon classificationAnimaliaHymenopteraHalictidae

Blüthgen, 1927

6B968EAC-9F7E-573D-A48F-098BEFC24ED7

Figures 28–33

Sphecodes chaprensis Blüthgen, 1927: 96–98, Fig. 30, ♂ (holotype: ♂, India, Chapra, Bengal, Mackenzie, B.M. TYPE HYM. 17a564; NHMUK 013380323; examined).

##### Diagnosis.

This species resembles *Sphecodes shillongensis* Blüthgen, 1927 and *S. simlaensis* Blüthgen, 1924, sharing a similar structure and sculpture of the body, including weakly developed antennal tyloids, a densely punctate mesoscutum and scarcely punctate metasomal terga. The species differs from *S. simlaensis* in having dense facial pubescence obscuring integument above the antennal sockets; from *S. shillongensis* it differs by shorter antennae with flagellomeres (from F3 onward) ca. 1.1–1.2 times as long as wide (versus 1.3) and shape of the gonostylus with a large membranous part.

##### Descriptive notes.

**Figures 28–33.**
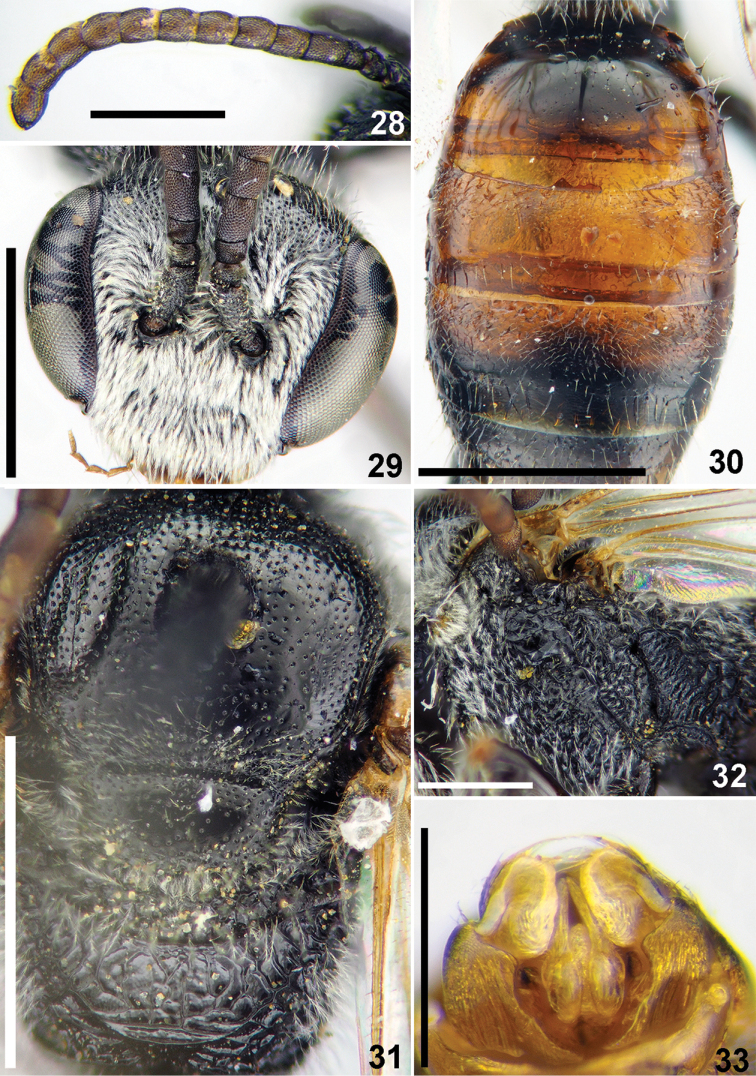
*Sphecodes chaprensis* Blüthgen, male **28** antennae, lateral view **29** head, frontal view **30** T1–T3, dorsal view **31** mesosoma, dorsal view **32** mesepisternum and hypoepimeral area, lateral view **33** genitalia, dorsal view. Scale bars: 1.0 mm (**29–31**), 0.5 mm (**28, 32, 33**).

Wings hyaline; hind wing with angle between basal (M) and cubital (Cu) veins almost 90°, costal margin with six or seven hamuli. Preoccipital carina absent. **Male.** Total body length 4.5–5.5 mm. Head transverse, ca. 1.15 times as wide as long (Fig. 29); vertex not elevated as seen in frontal view; antennae (Fig. 28) short, attaining posterior margin of mesoscutum, F1 0.7 times as long as wide, F2 1.3–1.4 times as long as wide, remaining flagellomeres ca. 1.1–1.2 as long as wide; tyloids weakly developed, at most semicircular across basal 1/4 of ventral surfaces of last flagellomeres (Fig. 28); ocello-ocular area with minute punctures separated by at most a puncture diameter; face above and below the antennal sockets with adpressed white pubescence obscuring integument. Gena with sparser pubescence. Mesoscutum and mesoscutellum (Fig. 31) finely punctate, sparser medially (15–20 μm / 0.5–3), becoming denser peripherally; mesepisternum and hypoepimeral area rugose (Fig. 32); propodeal triangle (metapostnotum) shining, with coarse longitudinal-winding wrinkles; remaining surfaces of propodeum rugose to reticulate-rugose. Metasomal terga scarcely punctate (Fig. 30); T1 almost impunctate, with a few minute punctures; remaining terga basally with tiny setae pores; marginal zones impunctate; T1 (apically) and T2 red, coloration of T3 variable; gonocoxite dorsally with impression; gonostylus as on Fig. 33. **Female** unknown.

##### Material examined.

Laos: 1 ♂, Phongsaly prov., 21°44'N, 102°12'E, Ban Natsa, 9–17.V.2004, 550 m, P. Pacholatko (OLBL/PCMS).

##### Distribution.

*Laos, India (Bihar).

#### Sphecodes discoverlifei

Taxon classificationAnimaliaHymenopteraHalictidae

Astafurova & Proshchalykin sp. nov.

0EB9116B-2E76-5672-BEE2-ACAD64C7C4BD

http://zoobank.org/94551AB3-C22B-4561-B1B7-B81C82C1D90A

Figures 34–35 , 36–42 , 43–45

##### Type material.

***Holotype***: ♂, laos, Phongsaly prov., Phongsaly env., 21°41'N, 102°06'E, 1500 m, 1–30.VI.2003, P. Pacholatko (PCMS), Fig. 42. ***Paratypes***: 2 ♂♂, the same label as for holotype, but VI.2003 (PCMS/ZISP); 2 ♀#, the same label as for holotype, but 28.V–20.VI.2003, V. Kuban (PCMS/ZISP).

##### Diagnosis.

Among the oriental species lacking a preoccipital carina and with five or six hamuli in hind wing the male of the new species is recognizable by having tyloids covering the entire ventral and lateral flagellar surfaces and also in the shape of the gonostylus which has an elongate membranous part; the female is similar to *Sphecodes tantalus* Nurse, 1903 by combination of the strongly transverse head, the metafemur strongly enlarged in proximal half, the mesoscutum with relatively sparse punctures, the impunctate T1, the narrow pygidial plate and the reddish metasoma, but it differs by not having an elevated vertex as seen in frontal view (in *S. tantalus* distance from top of head to upper margin of lateral ocellus approximately a lateral ocellar diameter).

##### Description.

Wings hyaline, weak yellowish with light brown stigma and yellowish veins; hind wing with the angle between basal (M) and cubital (Cu) veins 90°, costal margin with five hamuli. Preoccipital carina absent. **Female.** Total body length 6.0–6.5 mm (Fig. 35), fore wing 4.6–4.9 mm. Head black (Fig. 43); strongly transverse, ca. 1.3 times as wide as long; vertex not elevated as seen in frontal view; distance from top of head to upper margin of a lateral ocellus ca. two lateral ocellar diameters as seen in dorsal view; F1 0.8 times as long as wide, F2 0.9 times as long as wide, remaining flagellomeres 1.0–1.1 times as long as wide; labrum trapezoidal, 0.7 times as long as basal width; face densely punctate, with punctures separated by at most a puncture diameter; ocello-ocular area and gena with tiny setae pores (5–10 μm) separated by a few puncture diameters; face and gena with sparser pubescence, not obscuring integument. Mesosoma black, legs brownish with yellowish tarsi; mesoscutum and mesoscutellum (Fig. 44) with relatively sparse punctures (15–25 μm / 1–4) becoming denser peripherally; metafemur strongly enlarged in proximal half, maximum width 0.4 times its length; hypoepimeral area and mesepisternum rugose; propodeal triangle (metapostnotum) with coarse longitudinal wrinkles and shining interspaces (Fig. 44); lateral parts of propodeum coarsely reticulate-rugose. Metasomal T1 impunctate; remaining terga with a few minute setae pores (Fig. 45); marginal zones impunctate; T1–T4 mostly red, remaining terga red-brownish; pygidial plate dull, pointed apically, narrow, 0.6 times as wide as metabasitarsus. Sterna finely tessellate with dense shallow setae pores.

**Figures 34, 35.**
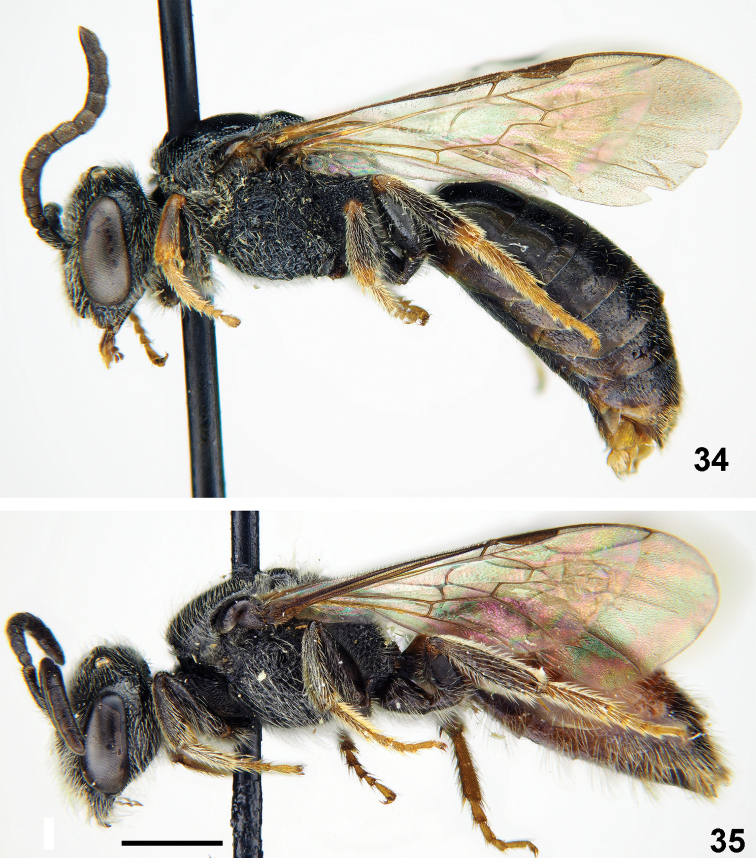
*Sphecodes discoverlifei* Astafurova & Proshchalykin, sp. nov. **34** holotype, male, lateral view **35** paratype, female, lateral view. Scale bars: 1.0 mm.

**Figures 36–42.**
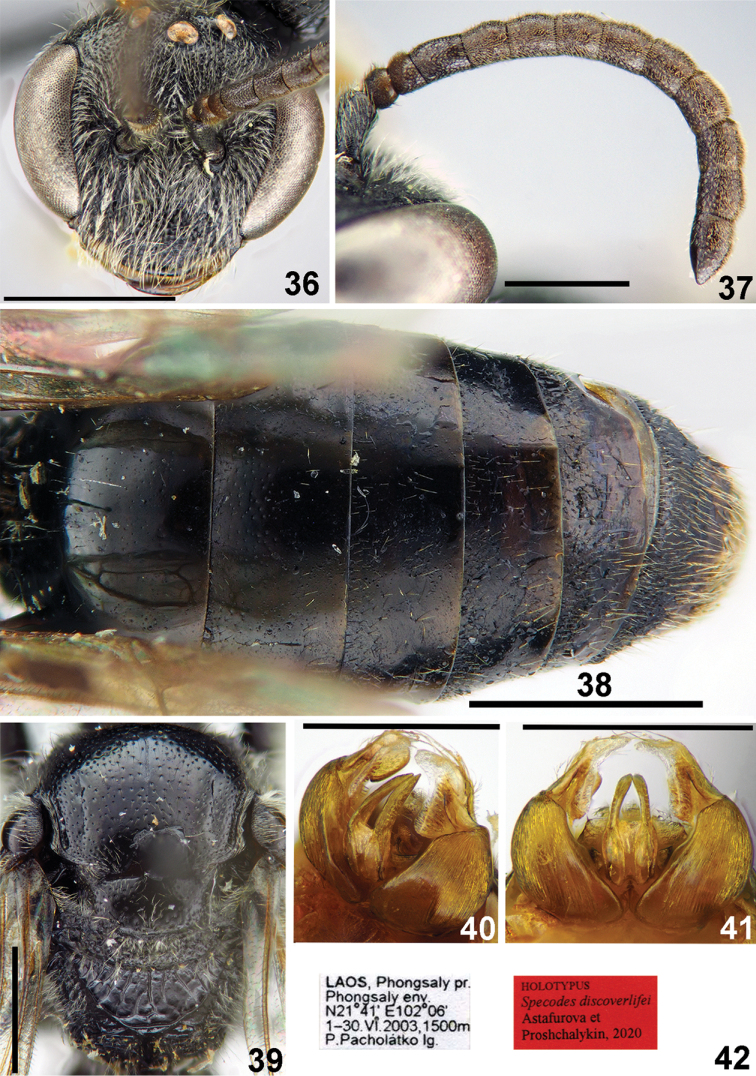
*Sphecodes discoverlifei* Astafurova & Proshchalykin, sp. nov., male, holotype **36** head, frontal view **37** antennae, lateral view **38** metasoma, dorsal view **39** mesosoma, dorsal view **40, 41** genitalia, dorso-lateral view (**40**), dorsal view (**41**) **42** labels. Scale bars: 1.0 mm (**36–39**), 0.5 mm (**40, 41**).

**Male.** Total body length 6–7 mm (Fig. 34), fore wing 4.5–5.0 mm. Head black (Fig. 36), transverse, ca. 1.2 times as wide as long; vertex not elevated as seen in frontal view and distance from top of head to upper margin of a lateral ocellus approximately two lateral ocellar diameters as seen in dorsal view; antenna short (Fig. 37), reaching posterior half of mesoscutum, F1 0.6 times as long as wide, F2 1.3–1.4 times as long as wide, remaining flagellomeres 1.1–1.2 times as long as wide; tyloids covering entire ventral and lateral flagellar surfaces; face densely punctate, the punctures separated byat most a half puncture diameter; ocello-ocular area and gena more sparsely punctate with punctures separated by approximately a puncture diameter; face and gena with sparser pubescence, not obscuring integument. Mesosoma black, tibia (partially) and tarsi yellow; mesoscutum (Fig. 39) irregularly punctate, with confluent punctures peripherally and sparser medially (15–25 μm / 1–4); mesoscutellum coarsely punctate (20–40 μm) with punctures separated by at most a puncture diameter; hypoepimeral area and mesepisternum reticulate-rugose; propodeal triangle with coarse longitudinal wrinkles and shiny interspaces; lateral part of propodeum coarsely reticulate-rugose. Metasoma dark brownish (Fig. 38); terga almost impunctate with a few minute punctures; sterna with sparse setae pores; gonocoxite dorsally with impression; gonostylus with elongate membranous part (Figs 40, 41).

##### Etymology.

This species is dedicated to name of the website https://www.discoverlife.org (creators are J.S. Ascher and J. Pickering), in recognition of its contribution to knowledge of bee biodiversity.

##### Distribution.

Only known from the type locality in Laos.

**Figures 43–45.**
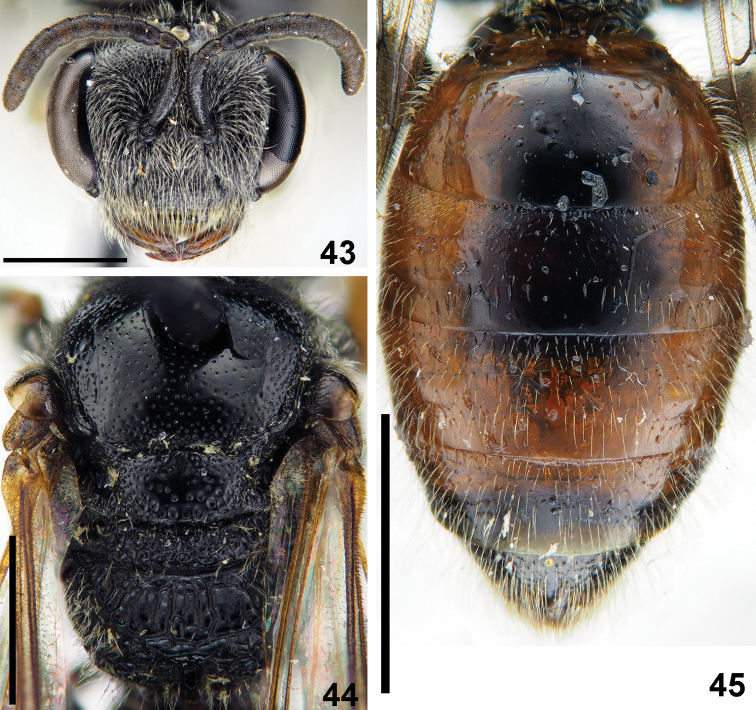
*Sphecodes discoverlifei* Astafurova & Proshchalykin, sp. nov., female, paratype **43** head, frontal view **44** mesosoma, dorsal view **45** metasoma, dorsal view. Scale bars: 1.0 mm.

#### Sphecodes distinctus

Taxon classificationAnimaliaHymenopteraHalictidae

Meyer, 1925

89605763-F008-55A3-840D-2292E81690C5

Figures 46–52

Sphecodes distinctus Meyer, 1925: 11, ♂ (holotype: ♂, Annam, Laos [Vietnam]; HNHM, examined, Fig. 52).

##### Diagnosis.

This species is similar to *Sphecodes formosanus* Cockerell, 1911 in having weakly developed tyloids, a densely punctate mesoscutum (close to areolate) and metasomal terga, but it differs in having a smaller body size (6.5–8.5 mm versus 9–10 mm), number of hamuli (seven or eight versus ten), and usually more developed tyloids (covering sometimes to basal 1/4–1/3 of distal flagellomeres compared to at most 1/5 in *S. formosanus*). Females of *S. takaensis*Blüthgen 1927, *S. howardi* and *S. binghami* are structurally and sculpturally close to the male of *S. distinctus* and it is possible that one of these is the unknown female for *S. distinctus*.

##### Descriptive notes.

**Figures 46–52.**
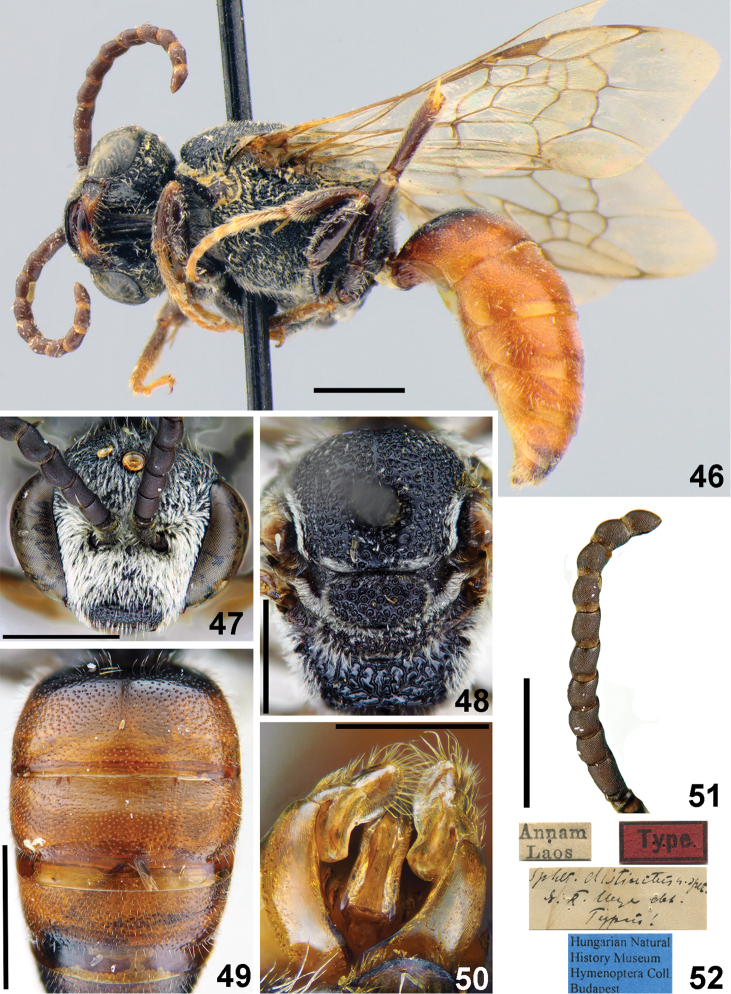
*Sphecodes distinctus* Meyer, male **46** Habitus, lateral view, holotype **47** head, frontal view **48** mesosoma, dorsal view **49** T1–T3, dorsal view **50** genitalia, dorsal view **51** antennae, lateral view **52** holotype labels. Scale bars: 1.0 mm (**46–49, 51**), 0.5 mm (**50**).

Wings with weak yellow-brownish darkening; hind wing with the angle between basal (M) and cubital (Cu) veins almost 80°, costal margin with seven or eight hamuli. Lateral preoccipital carina present. **Male.** Total body length 6.5–8.5 mm (Fig. 46). Head transverse (Fig. 47), ca. 1.2 times as wide as long; vertex elevated with distance from top of head to upper margin of lateral ocellus approximately an ocellar diameter as seen in frontal view; antennae (Fig. 51) reach posterior margin of mesoscutum, F2 1.6–1.7 times as long as wide, remaining flagellomeres 1.2–1.3 times as long as wide; tyloids semicircular across basal 1/7–1/3 of flagellar surfaces; face and vertex finely areolate-punctate; face (below and above the antennal sockets) and gena with adpressed white pubescence obscuring integument. Mesoscutum and mesoscutellum coarsely and densely punctate (Fig. 48), mostly with confluent punctures, but medially with interspaces approximately a puncture diameter; propodeal triangle (metapostnotum) roughly reticulate-rugose. Metasomal terga (Fig. 49) coarsely punctate (20–25 μm / 1–3); marginal zone on T1 finely punctate (impunctate along posterior margin); remaining marginal zones impunctate; T1–T4 and T5 basally red; gonocoxite dorsally without impression; gonostylus as shown in Fig. 50. **Female** unknown.

##### Material examined.

Thailand: 13 ♂♂, Nan, MaeCharim NP Eingang, 18°36'N, 100°58'E, 10–24.V.2012, E. & J. Holzschuh (OLBL/PCMS).

##### Published records.

Meyer 1925: 11 (Vietnam); Ascher and Pickering 2019 (Vietnam).

##### Distribution.

*Thailand, Vietnam.

##### Remarks.

Annam (type locality of *S. distinctus*) is actually located in Vietnam, not Laos as Meyer (1925: 11) pointed out.

#### Sphecodes duplex

Taxon classificationAnimaliaHymenopteraHalictidae

Blüthgen, 1927

E8CE53FD-74F6-5258-A7BA-AE5121B12F1C

Figures 2 , 4 , 6 , 7

Sphecodes biroi Meyer, 1925 (nom. praeocc., nec S. biroi Friese, 1909): 11, ♀ (syntypes: 2 ♀♀, “Singapure [Singapore], Biró 1898 leg.”; HNHM).
Sphecodes duplex Blüthgen, 1927: 78, replacement name for S. biroi Meyer, 1925 (nec S. biroi Friese, 1909).

##### Diagnosis.

Structurally and sculpturally this species is extremely similar to the female of *Sphecodes bakeri* Cockerell, 1915, but it differs in the ocello-ocular area (Fig. 4)having shiny interspaces and T2 basally possessing fine and sparse punctures (versus ocello-ocular area with entirely confluent punctures without interspaces (Fig. 5) and T2 impunctate in *S. bakeri*).

##### Descriptive notes.

Wings with yellow-brownish darkening; hind wing with angle between basal (M) and cubital (Cu) veins almost 90°, costal margin with eight hamuli. Lateral preoccipital carina present. **Female.** Total body length 5–6 mm. Head strongly transverse (Fig. 2), ca. 1.35 times as wide as long; vertex weakly elevated with distance from top of head to upper margin of lateral ocellus approximately a lateral ocellar diameter as seen in frontal view; labrum trapezoidal, 0.6 times as long as basal width; face and ocello-ocular area with dense punctures separated by at most a half puncture diameter; paraocular and supraclypeal areas with adpressed white pubescence obscuring integument, gena with sparser pubescence. Mesoscutum and mesoscutellum (Fig. 6) areolate-punctate (30–50 μm); propodeal triangle (metapostnotum) with longitudinal wrinkles and deep large shining interspaces between them; lateral parts of propodeum with parallel wrinkles and large shining interspaces; mesepisternum reticulate-rugose. Metasomal terga red, almost impunctate (Fig. 7); T2 basally with sparse and minute (ca. 5 μm) punctures; pygidial plate 1.2 times as wide as metabasitarsus. **Male** unknown.

##### Material examined.

Indonesia: 1 ♀, Java, Buitzorg [*S. duplex* Blüthgen det.] (ZMHB); Malaysia: 1 ♀, Malaya, Kuala Sleh, jungle, 15.III.1936, H.T. Pagden (NHMUK 013380358).

##### Published records.

Meyer 1925: 11 (Singapore); Blüthgen 1927: 78 (Indonesia); Ascher and Pickering 2019 (Singapore).

##### Distribution.

Indonesia, *Malaysia, Singapore.

##### Remarks.

This species is probably the unknown female of *S. samarensis.*

#### Sphecodes engeli

Taxon classificationAnimaliaHymenopteraHalictidae

Astafurova & Proshchalykin sp. nov.

F4AF45AD-C547-5D98-B6BC-1923EFBF8510

http://zoobank.org/B2D1E20B-86FF-42AE-AE78-37A4F9EEF2D2

Figures 53 , 54–59

##### Type material.

***Holotype***: ♀, Laos, Hua Phan Prov., Ban Saleui, Phou Pan Mts., 20°13'30"N, 103°59'26"E, 1350–1900 m, 08.V.2012, C. Holzschuh & locals (OLBL), Fig. 59. ***Paratypes***: 1 ♀, the same label as for holotype, but 27–28.IV.2011 (OLBL); Vietnam: 1 ♀, prov. Hoa Binh, Pa Co, 27–28.IV.2002, S. Belokobylskij (ZISP).

##### Diagnosis.

As with members of the Palaearctic *hyalinatus* species group (Astafurova and Proshchalykin 2017a), the new species has a pronotum rounded between the dorsal and lateral surfaces, but it differs in the strongly transverse head (1.3 times as wide as long) with a straight upper margin as seen in frontal view (versus head 1.2–1.25 times as wide as long with rounded vertex as seen in frontal view in species of the *hyalinatus* group).

##### Description.

**Figure 53.**
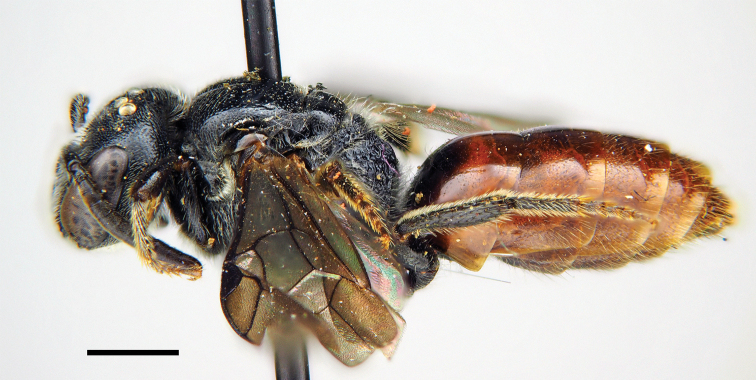
*Sphecodes engeli* Astafurova & Proshchalykin, sp. nov., female, holotype, lateral view. Slale bars: 1.0 mm.

**Figures 54–59.**
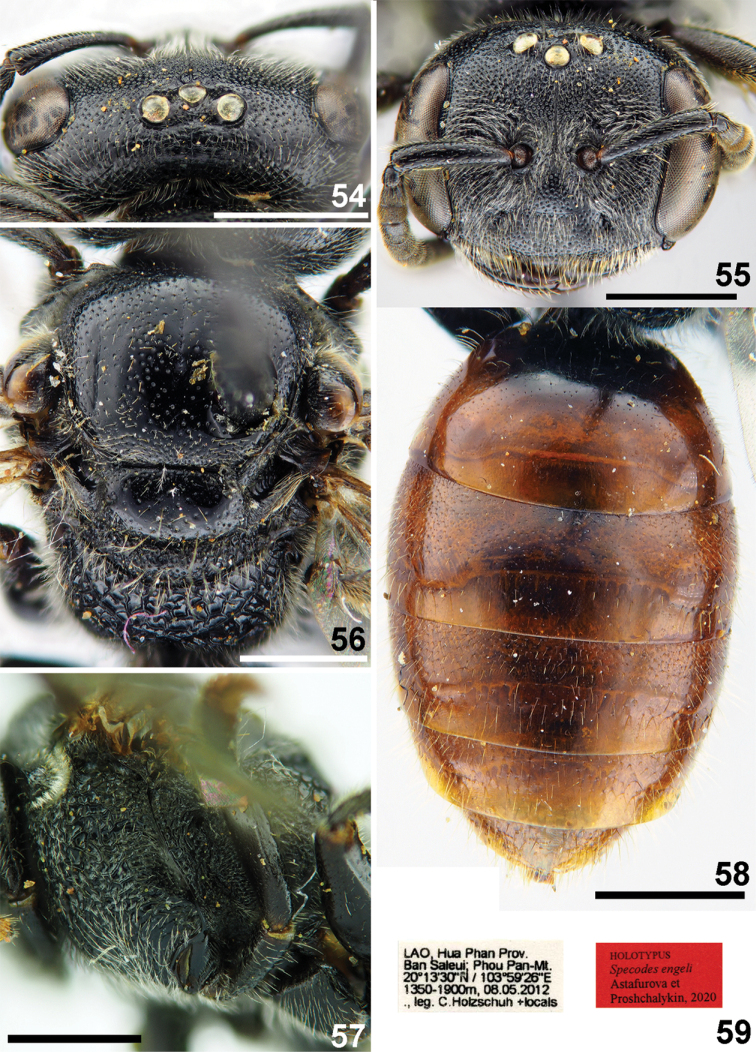
*Sphecodes engeli* Astafurova & Proshchalykin, sp. nov., female, holotype **54, 55** head, dorsal view (**54**), frontal view (**55**) **56, 57** mesosoma, dorsal view (**56**), lateral view (**57**) **58** metasoma, dorsal view **59** labels. Scale bars: 1.0 mm.

Wings with weak brownish darkening, veins and stigma brown; hind wing with angle between basal (M) and cubital (Cu) veins ca. 90°, costal margin with six hamuli. Preoccipital carina absent. **Female.** (holotype. Fig. 53). Total body length 7.5–8.5 mm, fore wing 6.5–7.0 mm. Head black (Fig. 55); strongly transverse, ca. 1.3 times as wide as long; vertex weakly elevated as seen in frontalview, distance from top of head to upper margin of a lateral ocellus approximately a half lateral ocellar diameter as seen in frontal view and ca. 2 diameters as seen in dorsal view; mandible bidentate; labrum trapezoidal, 0.7 times as long as basal width; gena wide, 1.2 times as wide as eye; clypeus medially slightly emarginated; supraclypeal area weakly bulging; clypeus and supraclypeal area with punctures (15–25 μm) separated by at most a puncture diameter; paraocular area and frons with confluent punctures, ocello-ocular area with punctures separated by 1–3 puncture diameters (Fig. 54), vertex behind ocelli and gena strigose; face below antennal sockets with sparse plumose setae, gena with sparse thin setae. Mesosoma black (Fig. 56); pronotum rounded between the dorsal and lateral surfaces; mesoscutum finely punctate (15–25 μm / 1–4); mesoscutellum irregularly punctate, medially sparsely; metafemur enlarged in proximal half, maximum width 0.4 times its length; hypoepimeral area, mesepisternum, propodeal triangle (metapostnotum) and lateral parts of propodeum reticulate rugose (Figs 56, 57). Mesosoma (Fig. 58) sparsely punctate, T1 impunctate or with a few fine punctures; remaining terga basally with sparse and fine punctures (5–10 μm); marginal zones impunctate; pygidial plate dull, pointed apically, narrow, 0.6–0.7 times as wide as metabasitarsus; T1–T4 red, remaining terga red or red-brownish; sterna tessellate, with tiny and sparse shallow setae pores on S2 and coarse and dense on S3–S5.

**Male** unknown.

##### Etymology.

This species is dedicated to our colleague Dr. Michael S. Engel (University of Kansas, USA), in recognition of his significant contributions to systematic entomology.

##### Distribution.

Laos, Vietnam.

#### Sphecodes fumipennis

Taxon classificationAnimaliaHymenopteraHalictidae

Smith, 1853

DFEE4DF2-F5E2-5057-9E10-9233D24B1068

Figures 60–63 , 64–68

Sphecodes fumipennis Smith, 1853: 36, ♀ (holotype: ♀, N. India, coll. J.S. Baly; NHMUK, not examined).

##### Diagnosis.

The male of this species resembles *Sphecodes assamensis* Blüthgen, 1927 in having a similar size of antennal tyloids and shape of the gonostylus, but it differs from this species by shining interspaces on vertex and mesoscutum (dull in *S. assamensis*), and the number of hamuli (ten or twelve versus eight). The female differs from other oriental species by combination of the following characters; lack of a preoccipital carina, large body length (9.5–12 mm), eleven or twelve hamuli, mesoscutum mostly punctate-areolate and vertex strongly elevated. With these characteristics the female is similar to the palaearctic *S. albilabris* (Fabricius, 1793), but it differs in a sparsely punctate T1 disc with punctures separated by 2–6 puncture diameters (versus 0.5–2 in *S. albilabris*).

##### Descriptive notes.

**Figures 60–63.**
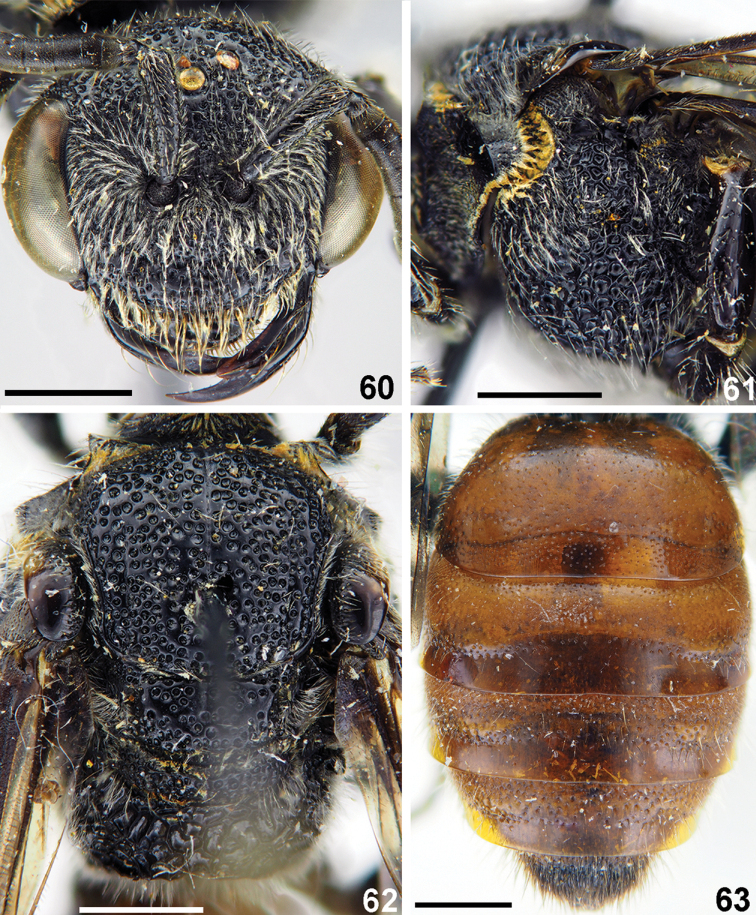
*Sphecodes fumipennis* Smith, female **60** head, frontal view **61, 62** mesosoma, lateral view (**61**), dorsal view (**62**) **63** metasoma, dorsal view. Scale bars: 1.0 mm.

**Figures 64–68.**
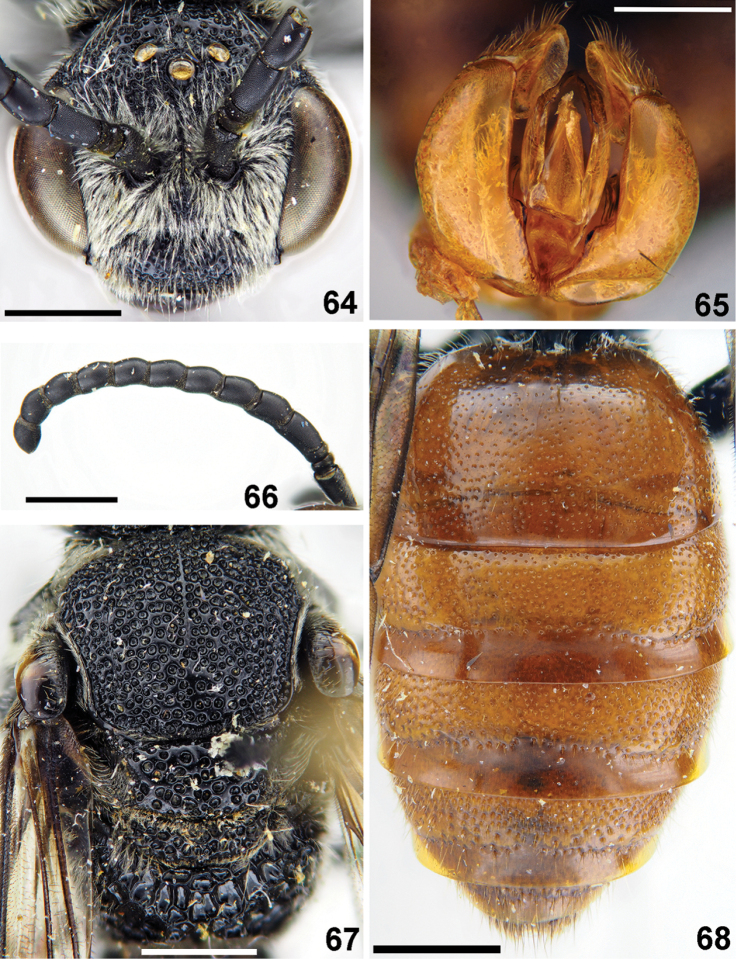
*Sphecodes fumipennis* Smith, male **64** head, frontal view **65** genitalia, dorsal view **66** antennae, lateral view **67** mesosoma, dorsal view **68** metasoma, dorsal view. Scale bars: 1.0 mm (**64, 66–68**), 0.5 mm (**65**).

Wings with strong brownish darkening; hind wing with the angle between basal (M) and cubital (Cu) veins ca. 70°, costal margin with eleven or twelve hamuli. **Female.** Total body length 9.5–12 mm. Head (Fig. 60) transverse, 1.25 times as wide as long; vertex strongly elevated with distance from top of head to upper margin of lateral ocellus ca. two lateral ocellar diameters as seen in frontal view; supraclypeal area swollen; labrum short, semi-oval, 0.45 times as long as basal width; ocello-ocular area areolate-punctate, but vertex behind ocelli with shiny interspaces; paraocular areas and gena with relatively dense plumose pubescence although not obscuring integument. Mesoscutum and mesoscutellum (Fig. 62) mostly with confluent punctures (50–75 μm), but medially with a few interspaces at most 1–2 puncture diameters; propodeal triangle (metapostnotum) coarsely reticulate-rugose; mesepisternum (Fig. 61) areolate on an upper half to reticulate below. Metasoma (Fig. 63) red; T1 sparsely punctate (ca. 25 μm / 2–6), finer and denser on marginal zone; remaining terga more densely and coarsely punctate, but marginal zones impunctate; pygidial plate dull, 1.3–1.4 as wide as metabasitarsus. **Male.** Total body length 9.5–12 mm. Head (Fig. 64) transverse, 1.2 times as wide as long; vertex strongly elevated with the distance from top of head to upper margin of lateral ocellus more than two lateral ocellar diameters as seen in frontal view; antenna (Fig. 66) long, reaching mesoscutellum, F2 1.8 times as long as wide, remaining flagellomeres ca. 1.4–1.5 times as long as wide; tyloids weakly developed, narrowly semicircular across basal 1/8–1/7 of flagellar surfaces and narrowly linear across remainder of flagellomere as seen in lateral view. Mesoscutum and mesoscutellum (Fig. 67) mostly areolate-punctate, but medially with a few interspaces approximately a puncture diameter wide; propodeum and mesepisternum as in the female. Metasoma (Fig. 68) coarsely and densely punctate, sparser on T1 (25–35 μm / 0.5–3); marginal zones impunctate except on T1; gonocoxite dorsally without impression; gonostylus (Fig. 65) short, with small membranous part.

##### Material examined.

Laos: 2 ♀♀, 1 ♂, Louang Phrabang pr., Ban Song Cha, 1200 m, V. 1999, V. Kuban (OLBL/PCMS).

##### Published records.

Blüthgen 1924: 489 (Myanmar).

##### Distribution.

*Laos, Myanmar, India (Sikkim).

#### Sphecodes howardi

Taxon classificationAnimaliaHymenopteraHalictidae

Cockerell, 1922

7DF37BEA-4D20-50F2-B18B-3D296586142A

Figures 69 , 71

Sphecodes howardi Cockerell, 1922: 12, ♀ (holotype: ♀, Canton [= Guangdong], China, C.W. Howard Collection, Type No 24885USNM; USNM, http://n2t.net/ark:/65665/3129f6c5d-578d-446f-bdc7-f59ccb6213e0).

##### Diagnosis.

This species is most close to *Sphecodes kershawi* and also resembles *S. formosanus* Cockerell, 1911, *S. takaensis* Blüthgen, 1927, and *S. binghami* owing to similar structure, sculpture, and coloration of the body, including a densely punctate disc and marginal zone of T1 (differences between females of these species are outlined in Table 2). Structurally and sculpturally the species is also close to *S. distinctus* and *S. sibuyanensis* Cockerell, 1925 and one of these species may possibly be the unknown male of *S. howardi*, especially *S. sibuyanensis* which has the same brown wing coloration (lighter and yellowish in *S. distinctus*).

**Table 2.** Differences between females of *Sphecodes howardi*, *S. formosanus*, *S. takaensis*, *S. binghami*, and *S. kershawi*.

Characters	*Sphecodes* species				
	* howardi *	* formosanus *	* takaensis *	* binghami *	* kershawi *
F3	About as long as wide	About as long as wide	0.7–0.8 times as long as wide	About as long as wide	About as long as wide
Mesoscutum	With coarse and confluent punctures, but medially with interspaces 0.5–1.0 puncture diameter	Densely punctate with punctures separated at most a puncture diameter	With coarse and confluent punctures, but medially with interspaces 0.5–1.0 puncture diameter	With coarse and confluent punctures, but medially with interspaces 0.5–1.0 puncture diameter	Mostly areolate-punctate
T4 marginal zone	Tessellate	Smooth	Smooth	Smooth	Smooth or unclearly tessellate
Pygidial plate	Narrower than metabasitarsus	As wide as metabasitarsus	Narrower than metabasitarsus	As wide as metabasitarsus	Narrower than metabasitarsus
Number of hamuli	7–8	9–10	8	8–9	6–7
Distribution	Malaysia, Myanmar, China (Guangdong)	China (Taiwan)	China (Taiwan)	Malaysia, Myanmar	Indonesia, Malaysia, Myanmar, Thailand, China (Macao)

##### Descriptive notes.

**Figures 69–74.**
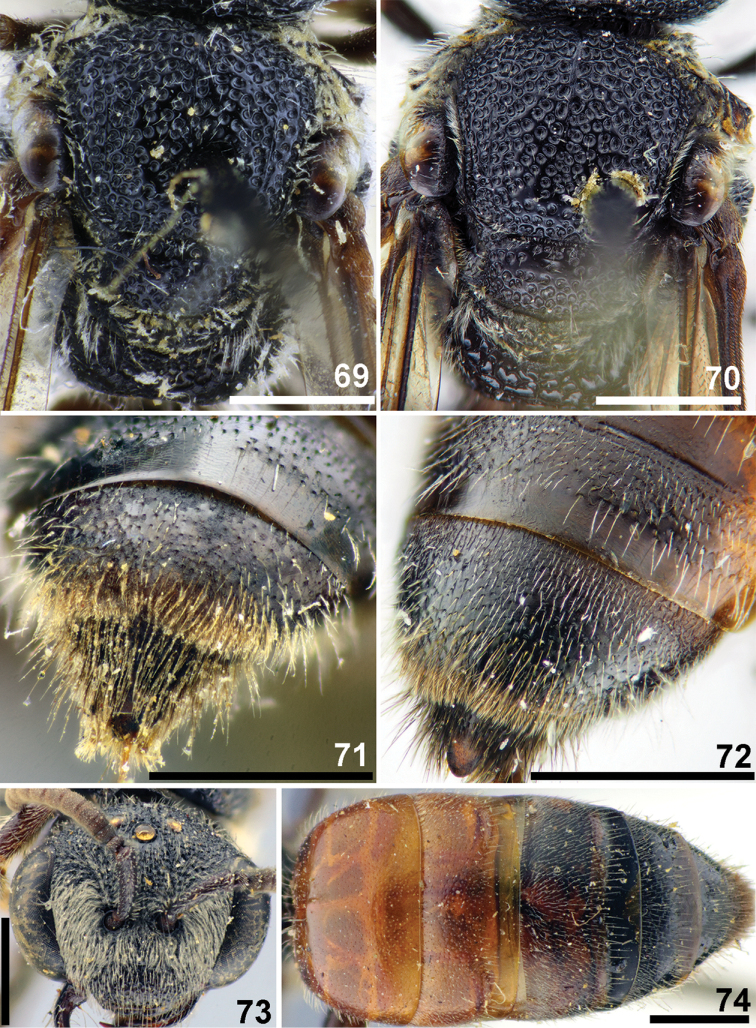
*Sphecodes howardi* Cockerell (**69, 71**), *S. kershawi* Perkins (**70, 72–74**), females **69, 70** mesosoma, dorsal view **71, 72** T4–T6, dorso-lateral **73** head, frontal view **74** metasoma, dorsal view. Scale bars: 1.0 mm.

Wings with brownish darkening; hind wing with angle between basal (M) and cubital (Cu) veins ca. 70°, costal margin with seven or eight hamuli. Lateral preoccipital carina present. **Female.** Total body length 8.5–9.5 mm. Head transverse, ca. 1.25 times as wide as long; vertex elevated with distance from top of head to upper margin of lateral ocellus approximately a lateral ocellar diameter as seen in frontal view; labrum semi-oval, 0.4 times as long as basal width; face and vertex areolate-punctate; paraocular (below and above the antennal sockets), supraclypeal areas and gena with adpressed white pubescence obscuring integument. Mesoscutum and mesoscutellum mostly with areolate punctures (40–75 μm), but medially with a few shining interspaces of approximately a puncture diameter (Fig. 69); propodeal triangle (metapostnotum) reticulate-rugose (sculpture formingone or two rows of large deep cells); mesepisternum reticulate-rugose. Metasomal T1 on disc and marginal zone finely and densely punctate (10–15 μm / 0.5–3), remaining terga similarly punctate, but with impunctate marginal zones; T4 marginal zone finely tessellate (Fig. 71); T1–T3 red, T4 variable; pygidial plate 0.7 times as wide as metabasitarsus. **Male** unknown.

##### Material examined.

Malaysia: 1 ♀, Titi Serong Perak, 29.III.1930, H.T. Pagden (NHMUK 013380439); Myanmar: 1 ♀, Upper Burma, Nam Tamai Valley, 3000 ft, 12.VIII.1938, R. Kaulback, 27°42'N, 97°54'E (NHMUK 013380337); China: 1 ♀, Canton, 1916–1918, H. Weigold (ZMHB).

##### Distribution.

*Malaysia, *Myanmar, China (Guangdong).

#### Sphecodes ilyadadaria

Taxon classificationAnimaliaHymenopteraHalictidae

Astafurova sp. nov.

67F18E02-B3C8-5429-A230-0D0B7EB828AC

http://zoobank.org/281ED4DF-FFBA-4341-A305-43A791BAD494

Figures 75 , 76–80

##### Type material.

**Figure 75.**
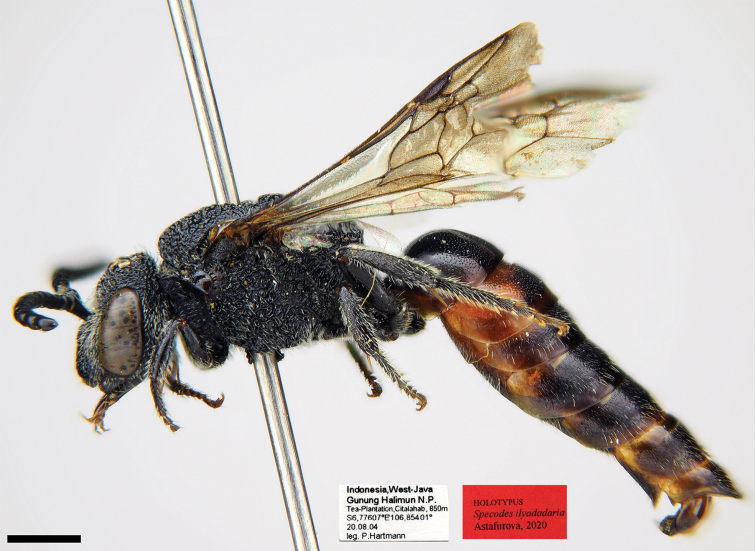
*Sphecodes ilyadadaria* Astafurova, sp. nov., male, holotype, lateral view. Scale bars: 1.0 mm.

***Holotype***: ♂, indonesia, West Java, Gunung Halimun N.P., Tea Plantation, Citalahab, 850 m, 6.77607N, 106.85401E, 20.VIII.2004, P. Hartmann (ZSM), Fig. 75. ***Paratype***: 1 ♂, the same label as for holotype (ZMS). Additional material (thisspecimen was determined as belonging the new species by photos and descriptive notes): 1 ♂, NE Sulawesi, 47 km WSW Kotamobagu, Dumoga-Bone N.P., Toraut (forest edge), 211 m, V.1985, G.R. Else, NHMUK 013380345 [aff. insularis Astafurova det. 2019].

##### Diagnosis.

The new species most closely resembles *Sphecodes insularis* Smith, 1858, from which it differs by having an areolate punctate mesoscutum (with interspaces between punctures up to a puncture diameter in *S. insularis*).

##### Description.

**Figures 76–80.**
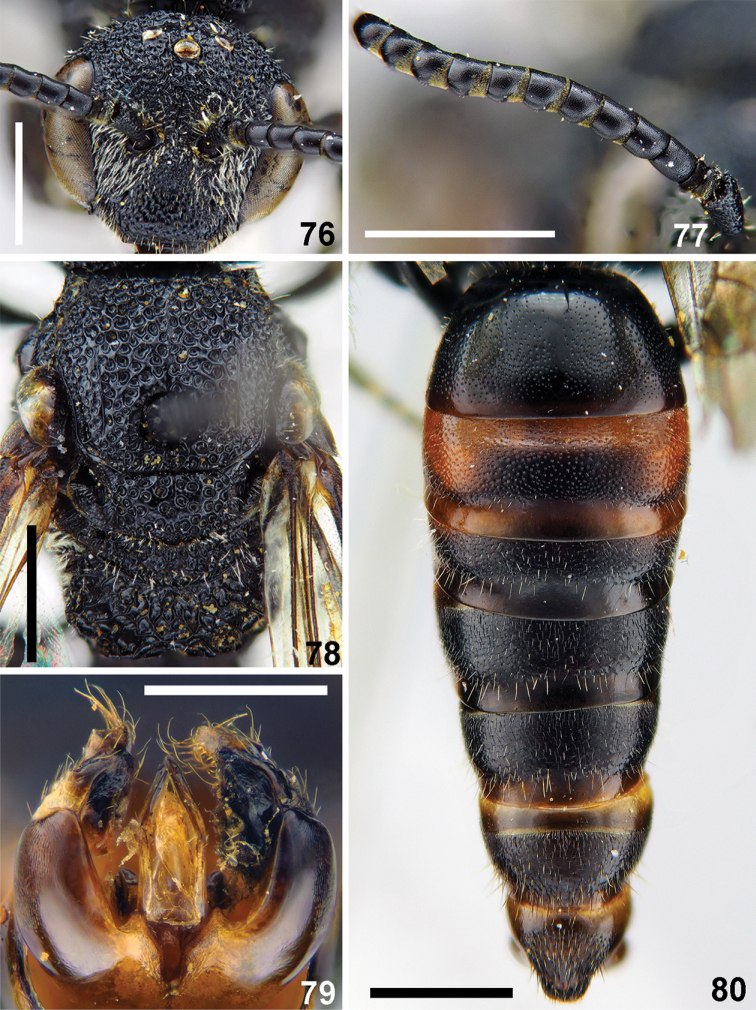
*Sphecodes ilyadadaria* Astafurova, sp. nov., male, holotype **76** head, frontal view **77** antennae, lateral view **78** mesosoma, dorsal view **79** genitalia, dorsal view **80** metasoma, dorsal view. Scale bars: 1.0 mm (**76–78, 80**), 0.5 mm (**79**).

Wings with weak yellow-brownish darkening, veins and stigma brown; hind wing with the angle between basal (M) and cubital (Cu) veins ca. 70°, costal margin with seven or eight hamuli. Lateral preoccipital carina well developed. **Male.** (holotype, Fig. 75). Total body length 8.5–9.0 mm, fore wing 5.6–5.7 mm. Head black (Fig. 76); weakly transverse, ca. 1.15 times as wide as long; vertex elevated, distance from top of head to upper margin of a lateral ocellus ca. one and a half of lateral ocellar diameter as seen in frontal view and ca. two as seen in dorsal view; antenna short (Fig. 77), reaching posterior half of mesoscutum; F1 strongly transverse, 0.4 times as long as wide; remaining flagellomeres 1.2–1.3 times as long as wide; tyloids semi-oval across at most basal 1/2 of last flagellomeres; supraclypeal area weakly bulging; clypeus shining, densely punctate with the punctures (20–30 μm) separated by at most a half puncture diameter. Supraclypeal and paraocular areas dull, finely areolate-punctate (15–25 μm), but frons and vertex close to reticulate-rugose; gena shining, rugose with sparse short setae; paraocular and supraclypeal areas with dense plumose adpressed pubescence. Mesosoma black (Fig. 78); mesoscutum coarsely areolate-punctate (50–75 μm), medially closer to reticulate-rugose; mesoscutellum densely and coarsely punctate, medially with the punctures separated by at most a puncture diameter; hypoepimeral area reticulate rugose; mesepisternum and propodeal triangle (metapostnotum) roughly reticulate-rugose; mesepisternum with sparse and thin short setae; lateral parts of propodeum shining, close to striate. Metasoma (Fig. 80) distinctly punctate, T1 with minute (5–15 μm) numerous punctures; remaining terga coarsely punctate (15–25 μm / 0.5–2); marginal areas impunctate except on T1 which has fine and sparse punctures basally; sterna tessellate with shallow setae pores; gonocoxite dorsally without impression; gonostylus with triangular apical process (Fig. 79); T1–T3 and S1–S3 variable in coloration, partially red, remaining terga and sterna brownish.

**Female** unknown.

##### Etymology.

The species is named after the author’s daughter Darya Gayday and her husband Ilya Gayday, who recently married. It is to be treated as a noun.

##### Distribution.

Indonesia.

#### Sphecodes kershawi

Taxon classificationAnimaliaHymenopteraHalictidae

Perkins, 1921

E52EA215-D2F5-5357-BC5F-818C6D35992D

Figures 70 , 72–74 , 81–85

Sphecodes kershawi Perkins, 1921: 9, ♂ (syntypes: ♂♂, China, Macao, J.C. Kershaw leg.; NHMUK, ZMHB, examined).
Sphecodes javanensis Blüthgen, 1927: 69–70, ♂ (holotype: ♂, Indonesia, Java, Buitenzorg, VIII. 1920; NHMW, examined). Syn. nov.

##### Diagnosis.

The male of this species is closest to *Sphecodes sibuyanensis* Cockerell, 1925 owing to similar short antennae with tyloids covering the entire ventral flagellar surface, a densely punctate metasoma (including marginal zone on T1) and in the shape of the gonostylus, with an elongate membranous part. From *S. sibuyanensis* it differs by a mostly punctate-areolate mesoscutum (versus a lot of mesoscutellar puncturesseparated by 0.5–1 puncture diameter). The female is closest to *S. howardi* and is also similar to *S. formosanus*, *S. takaensis*, and *S. binghami* owing to a similar structure, sculpture and coloration of the body, including the densely punctate disc and marginal zone of T1 (differences between females of these species are outlined in Table 2).

##### Descriptive notes.

**Figures 81–85.**
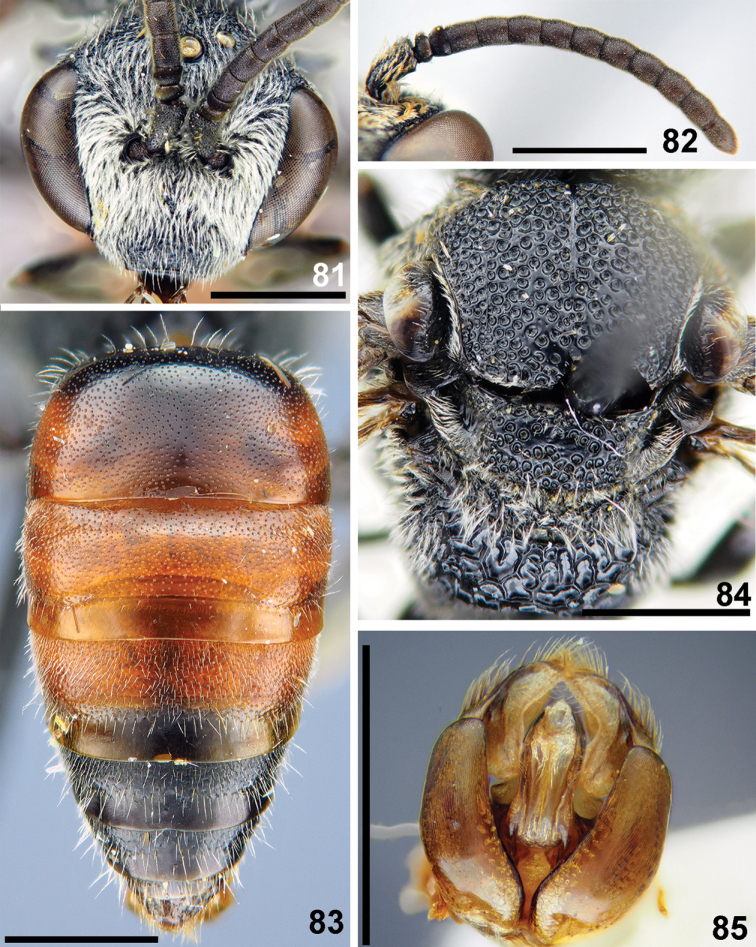
*Sphecodes kershawi* Perkins, male **81** head, frontal view **82** antennae, lateral view **83** metasoma, dorsal view **84** mesosoma, dorsal view **85** genitalia, dorsal view. Scale bars: 1.0 mm.

Wings with strong brownish darkening; hind wing with the angle between basal (M) and cubital (Cu) veins ca. 70°, costal margin with six, seven or eight hamuli. Lateral preoccipital carina present. **Female.** Total body length 7.5–8 mm. Head transverse (Fig. 73), ca. 1.2 times as wide as long; vertex elevated with distance from top of head to upper margin of lateral ocellus approximately a lateral ocellar diameter as seen in frontal view; labrum semi-oval, 0.5 times as long as basal width; face and vertex areolate-punctate; paraocular areas with dense adpressed white pubescence, gena with sparser pubescence not obscuring integument. Mesoscutum (Fig. 70) mostly areolate-punctate (50–75 μm) medially with a few punctures separated by at most 0.5–1 puncture diameter; mesoscutellum with confluent punctures and a few interspaces of approximately a puncture diameter. Propodeal triangle (metapostnotum) reticulate-rugose. Metasoma densely punctate (Fig. 74); T1 on disc and marginal zone finely punctate (10–15 μm / 0.5–3), remaining terga coarsely punctate (10–25 μm) with impunctate and smooth marginal zones, sometimes finely tessellate on T4 (Fig. 72); pygidial plate 0.7 times as wide as metabasitarsus; T1–T3 red. **Male.** Total body length 7–8 mm. Head transverse (Fig. 81), ca. 1.2 times as wide as long; vertex elevated with distance from top of head to upper margin of lateral ocellus approximately a lateral ocellar diameter as seen in frontal view; antennae short (Fig. 82), not reaching mesoscutellum, F1 0.6 times as long as wide, remaining flagellomeres ca. 1.2 times as long as wide, tyloids covering entire ventral flagellar surface. Mesosomal sculpture as in female (Fig. 84). Metasomal T1 densely punctate including marginal zone (10–20 μm / 0.5–2), remaining terga with impunctate marginal zones (Fig. 83); T1–T3 red or metasoma entirely black; gonocoxite dorsally without impression; gonostylus with elongate membranous part, apically with long setae (Fig. 85).

##### Material examined.

Indonesia: 1 ♂, Ceylon, Calutara, O.S. Wickwar, 1912-189 (NHMUK 013380334); Malaysia: 1 ♀, Malaya, Titi Serong Perak, 27.VIII.1931, H.T. Pagden (NHMUK 013380436); Myanmar: 1 ♀, Mali Hka Valley, Kachin Hills, 1500 ft, 15.XII.1930, F. Kingdon Ward., BM 1936-91 (NHMUK 013380338); Thailand: 1 ♀, 2 ♂♂, Nan, MaeCharim NPEingang, 18°36'N, 100°58'E, 206 m, 10–15.V.2012, E. & J. Holzschuh (OLBL/PCMS); China: 1 ♂ (syntype), Macao, J.C. Kershawi (ZMHB); 1 ♂ (syntype), Macao, J.C. Kershawi (NHMUK 013380474); 1 ♀, Macao, R.C.L. Perkins Coll., B.M. 1942-95 (NHMUK 013380470).

##### Published records.

Blüthgen 1927: 69 (Indonesia, as *S. javanensis*).

##### Distribution.

Indonesia, *Malaysia, *Myanmar, *Thailand, China (Macao).

#### Sphecodes laticeps

Taxon classificationAnimaliaHymenopteraHalictidae

Meyer, 1920

25BBFC8C-B830-5A3F-9741-75BA22945BBF

Figures 86–88 , 89–93

Sphecodes laticeps Meyer, 1920: 121, ♀, ♂ (lectotype (designated here): ♂, Formosa, Takao, H. Sauter S.G., 8.12.09 // Sphec. laticeps Meyer det. n. spec., !Type // Lectotypus, Sphecodes laticeps Meyer, 1920, design. Astafurova et al. 2020 <red label>; ZMHB); Paralectotypes: 2 ♀♀, Formosa, Taihorinsho, Sauter S.V., VIII. // Sphec. laticeps Meyer det. n. spec., Type, ZMHB, SDEI; 3 ♀♀, Taihorin, Formosa, H. Sauter, 1911 // 7.VI // Sphec. laticeps Meyer det. n. spec., Type; ZMHB.
Sphecodes candidius Meyer, 1925: 10, ♀ (holotype: ♀, Taiwan, “Lake Candidius 25./9/-10./10/ 1907”; HNHM). Synonymized by Blüthgen 1927: 85.
Sphecodes biroi mariae Cockerell, 1930: 162, ♂ (holotype: ♂; Thailand, “Siam, Nam, Jan. 8, 1928 (Cockerel)”; USNM, http://n2t.net/ark:/65665/3e3daca86-a75f-458d-b994-6723b995dccd). Syn. nov.

##### Diagnosis.

This species resembles *Sphecodes biroi* Friese, 1909 and *S. samarensis* Blüthgen, 1927 owing to a similar structure, sculpture and coloration of the body, including the shape of the male gonostylus. *S. laticeps* differs from *S. samarensis* by the shining and more elevated vertex with distance from top of head to upper margin of lateralocellus ca. one and a half or two lateral ocellar diameters as seen in frontal view (versus dull, areolate vertex with distance from top of head to upper margin of lateral ocellus half or one ocellar diameter. The female of *S. samarensis* is unknown, but these features are suitable for both sexes). The male of *S. laticeps* differs from *S. biroi* in having less developed tyloids and a glabrous spot on the ventral surfaces of flagellomeres (versus tyloids usually covering entire ventral flagellar surface or sometimes with small non-setae spot on basal flagellomeres). The females of *S. laticeps* and *S. biroi* are difficult to distinguish, but *S. laticeps* has T2 usually more distinctly punctate.

##### Descriptive notes.

**Figures 86–88.**
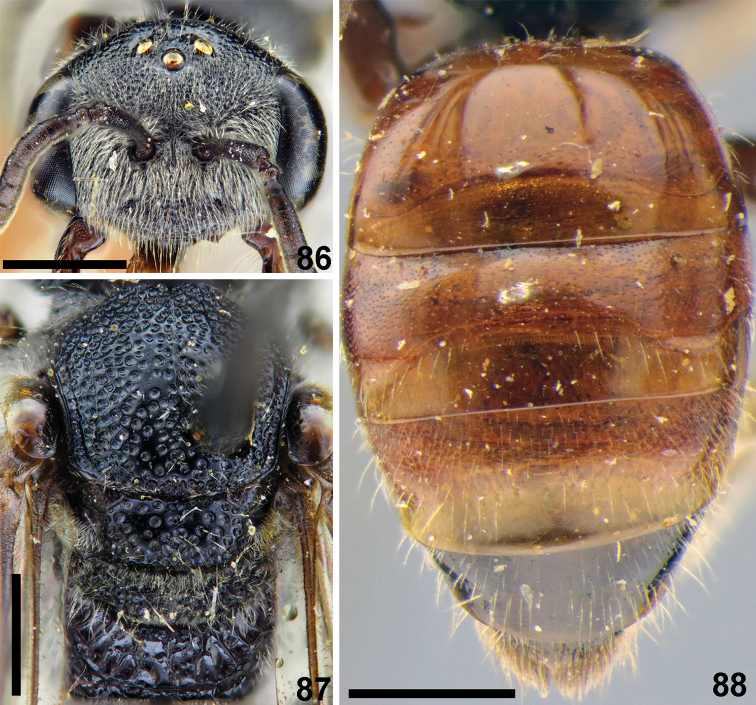
*Sphecodes laticeps* Meyer, female **86** head, frontal view **87** mesosoma, dorsal view **88** metasoma, dorsal view. Scale bars: 1.0 mm.

**Figures 89–93.**
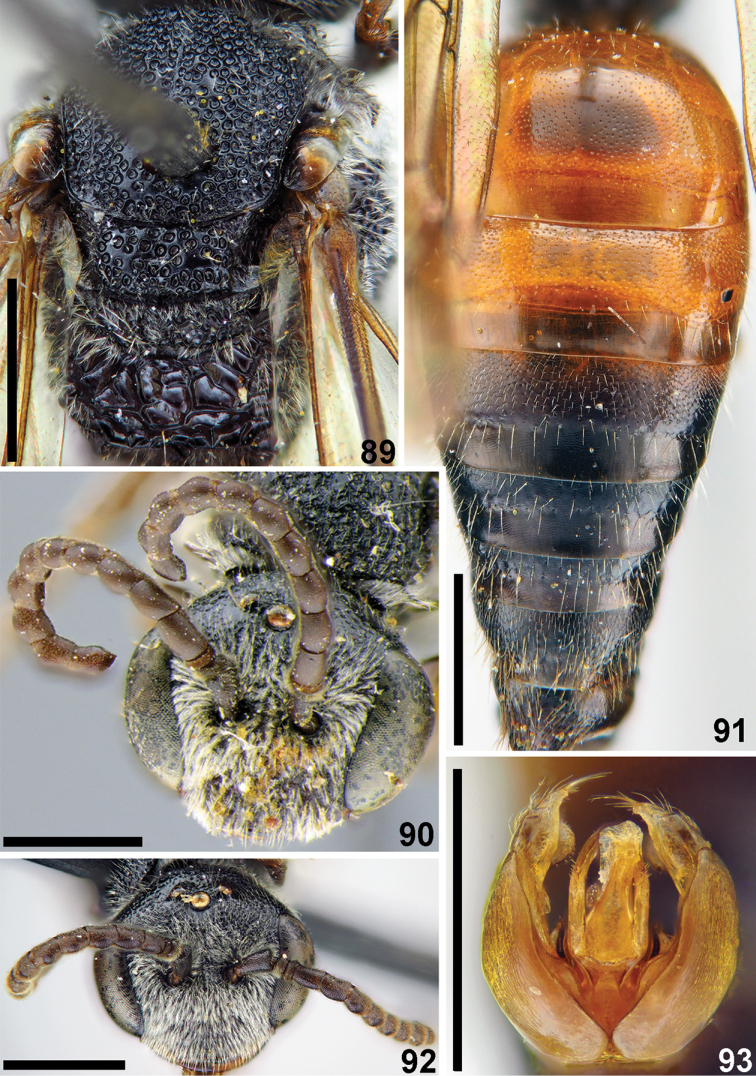
*Sphecodes laticeps* Meyer, male, lectotype (**90**) **89** mesosoma, dorsal view **90, 92** head, frontal view **91** metasoma, dorsal view **93** genitalia, dorsal view. Scale bars: 1.0 mm.

Wings with brownish darkening; hind wing with angle between basal (M) and cubital (Cu) veins almost 90°, costal margin with seven hamuli. Lateral preoccipital carina present. **Female.** Total body length 7–7.5 mm. Head strongly transverse (Fig. 86), ca. 1.3 times as wide as long; vertex elevated with distance from top of head to upper margin of lateral ocellus ca. one and a half of a lateral ocellar diameter as seen in frontal view; labrum trapezoidal, 0.6 times as long as basal width; ocello-ocular area shining with shallow punctures separated by 0.5–2 puncture diameters; face (below and above the antennal sockets) with adpressed white pubescence obscuring the paraocular and supraclypeal integuments, gena with sparser pubescence. Mesoscutum and mesoscutellum (Fig. 87) mostly with confluent punctures (30–40 μm) and medially with a few shining interspaces equal at most to one or two puncture diameters. Propodeal triangle (Fig. 87) roughly reticulate-rugose (sculpture forming one or two rows of large deep cells); mesepisternum reticulate-rugose. Metasomal T1 impunctate, T2 with minute and sparse punctures on medial part of disc, coarser and denser on lateral areas (10–15 μm / 2–4); marginal zones impunctate; pygidial plate as wide as metabasitarsus; T1–T3 red or darkish (Fig. 88). **Male.** Total body length 7–8 mm. Head transverse (Figs 90, 92), ca. 1.2 times as wide as long; vertex elevated with distance from top of head to upper margin of lateral ocellus ca. one and a half of a lateral ocellar diameter as seen in frontal view; antennae reach posterior margin of mesoscutum, F2 1.6–1.7 times as long as wide. Tyloids well developed, covering the entire lateral flagellar surfaces and peripheral part of ventral surface (with variable in size medial glabrous spot, Figs 90, 92). Mesoscutum (Fig. 89) mostly areolate-punctate, medially with a few shining interspaces equal at most to a puncture diameter. Propodeal triangle roughly reticulate-rugose; lateral parts of propodeum rugose with large smooth shining interspaces. Metasomal terga (Fig. 91) with minutely punctate (10–15 μm), variable in density; marginal zones impunctate; T1–T3 red; gonocoxite dorsally without impression; gonostylus as on Fig. 93.

##### Material examined.

Vietnam: 4 ♂♂, Gia Prov., Lai-Contum, Tran Lap, 20 km N Buon Luoi, 22–25.XI.1988, Sharkov (ZISP); 2 ♀♀, 50 km W Thanh Hoa, 9.I.1989, B. Korotyaev (ZISP); 1 ♀, Hanoi, 30.I.1989, Yanushev (ZISP).

##### Published records.

Cockerell 1930: 162 (Thailand, as *S. biroi mariae*); Ascher and Pickering 2019 (Thailand, as *S. biroi mariae*).

##### Distribution.

Thailand, *Vietnam, China (Taiwan).

#### Sphecodes montanus

Taxon classificationAnimaliaHymenopteraHalictidae

Smith, 1879

C7F121C3-8A26-51FC-A427-2EB5A408D37F

Figures 94–97 , 98–102

Sphecodes montanus Smith, 1879: 27, ♀, ♂ (syntype: ♀, Northern India, Masuri [Uttaranchal: Mussoorie], 7000 ft, B.M. Type HYM.17a549; NHMUK 013380316; examined).

##### Diagnosis.

This species is closest to *Sphecodes kozlovi* Astafurova & Proshchalykin, 2015 and *S. simillimus* Smith, 1873, both displaying a similar form to the male genitalia with a large membranous section of the gonostylus (Fig. 101), a similar size of antennal tyloids and a flat vertex with longitudinal carina (in the last feature, the species is also similar to *S. pieli* Cockerell, 1931). *S. montanus* differs from these three speciesby the possession of a weakly developed lateral preoccipital carina (absent in *S. kozlovi*, *S. simillimus* and *S. pieli*) and a narrower female pygidial plate which is 1.1–1.2 times as wide as metabasitarsus (versus 1.2–1.5).

##### Descriptive notes.

**Figures 94–97.**
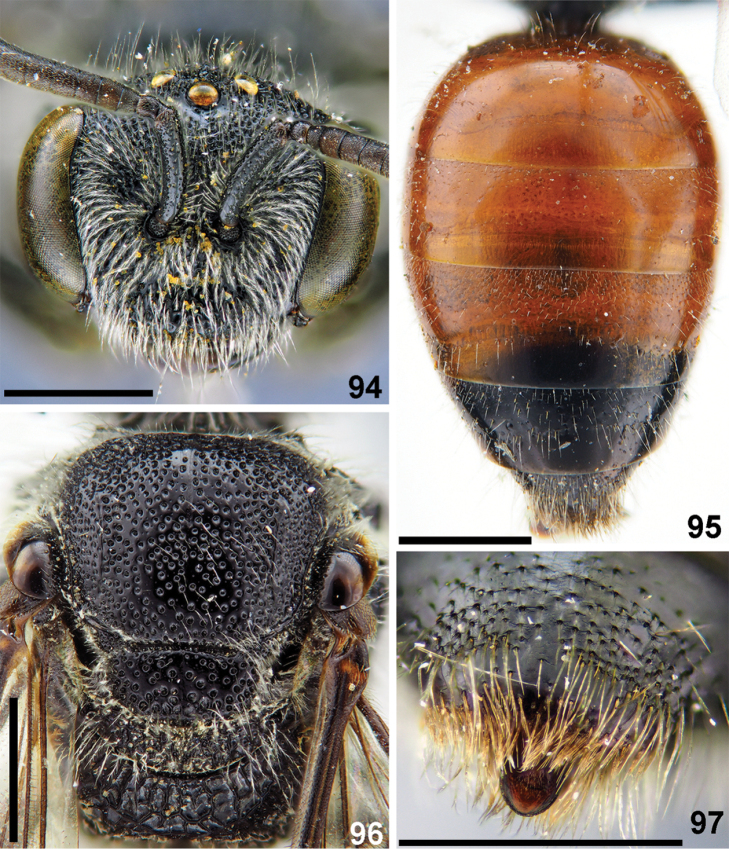
*Sphecodes montanus* Smith, female **94** head, frontal view **95** metasoma, dorsal view **96** mesosoma, dorsal view **97** T5–T6, pygidial plate, dorsal view. Scale bars: 1.0 mm (**94–96**), 0.5 mm (**97**).

**Figures 98–102.**
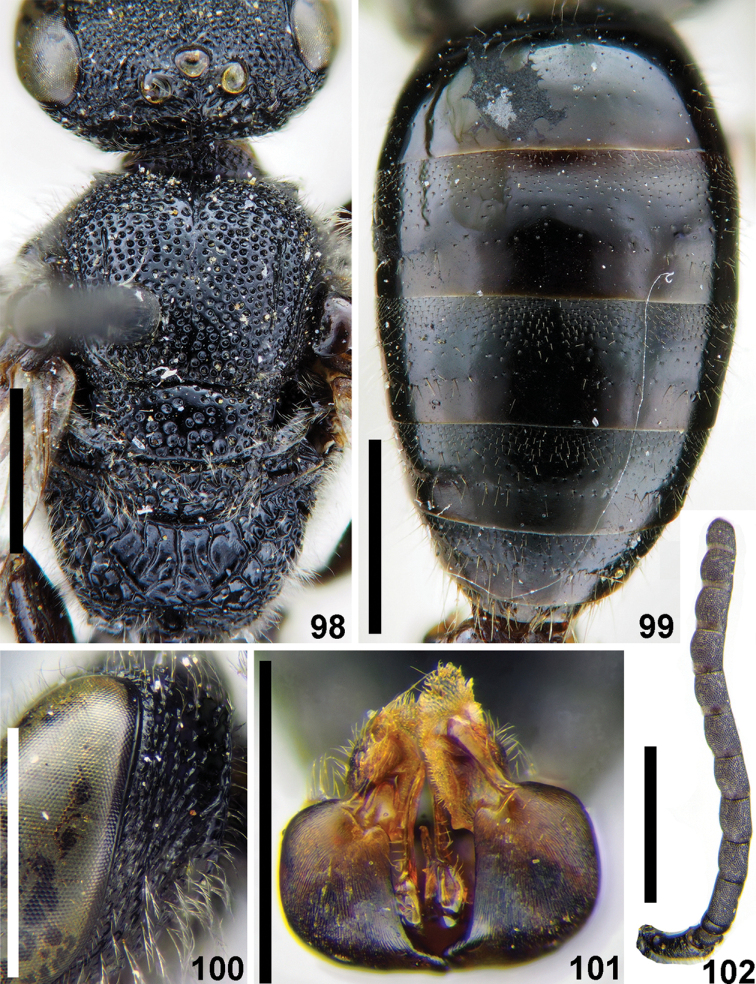
*Sphecodes montanus* Smith, male **98** head and mesosoma, dorsal view **99** metasoma, dorsal view **100** genal area and lateral preoccipital carina, lateral view **101** genitalia, dorsal view **102** antennae, lateral view. Scale bars: 1.0 mm.

Wings hyaline to weak brownish darkening; hind wing with the angle between basal (M) and cubital (Cu) veins ca. 90°, costal margin with five or six hamuli. Vertex with longitudinal carina; lateral preoccipital carina weakly developed (Fig. 100). **Female.** Total body length 7–8 mm. Head strongly transverse (Fig. 94), ca. 1.3 times as wide as long; vertex not elevated as seen in frontal view; labrum semi-oval, 0.5 times as long as basal width; face and ocello-ocular area areolate-punctate; paraocular areas and gena with sparse pubescence. Mesoscutum and mesoscutellum (Fig. 96) densely punctate with punctures separated by at most one or two puncture diameters, becoming denser (confluent) peripherally. Propodeal triangle (metapostnotum), mesepisternum and hypoepimeral area reticulate-rugose. Metasomal T1 impunctate, T2–T4 basally with sparse minute (5–10 μm) punctures (Fig. 95); marginal zones impunctate. Pygidial plate dull, 1.1–1.2 times as wide as metabasitarsus (Fig. 97); T1–T3 red. **Male.** Total body length 7–7.5 mm. Head transverse, ca. 1.2 times as wide as long; vertex not elevated as seen in frontal view; tyloids weakly developed, semi-oval, covering (at least from F4 onward) approximately basal 1/5–1/3 of flagellomeres (Fig. 102); F2 ca. 1.8 times as long as wide. Face and ocello-ocular area areolate-punctate. Mesosomal sculpture as in female (Fig. 98). Metasoma dark (Fig. 99); T1 impunctate; remaining terga basally finely and densely punctate. Gonocoxite dorsally without impression; gonostylus large, rectangular, apically with long setae (Fig. 101).

##### Material examined.

Laos: 1 ♂, Prov. Hua Phan, Phou Pan, Umg. Ort Ban Saleui, 20°13'N, 103°59'E, 1350–1900 m, 10–14.V.2012, C. Holzschuh & locals (OLBL/PCMS).

##### Distribution.

*Laos, India (Uttaranchal Rajasthan).

#### Sphecodes pseudoredivivus

Taxon classificationAnimaliaHymenopteraHalictidae

Astafurova & Proshchalykin sp. nov.

3306225D-FDCB-51B9-830A-46F4284107C9

http://zoobank.org/1D5A1DFD-7108-4F86-8AD7-3CB1F1CCB98B

**Figure 103.**
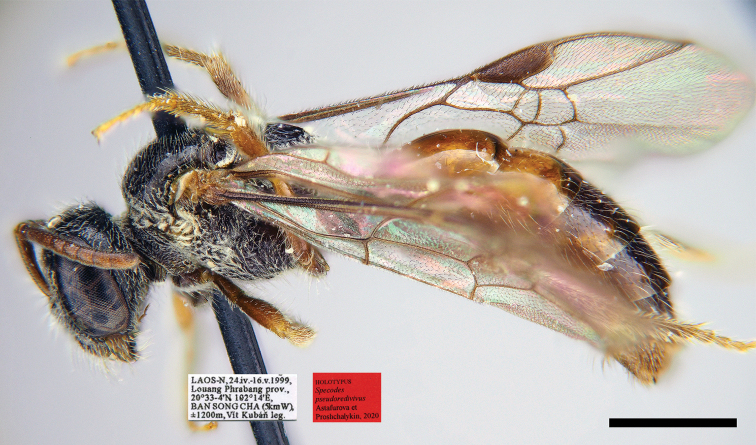
*Sphecodes pseudoredivivus* Astafurova & Proshchalykin, sp. nov., female, holotype, lateral view. Scale bars: 1.0 mm.

**Figures 104–109.**
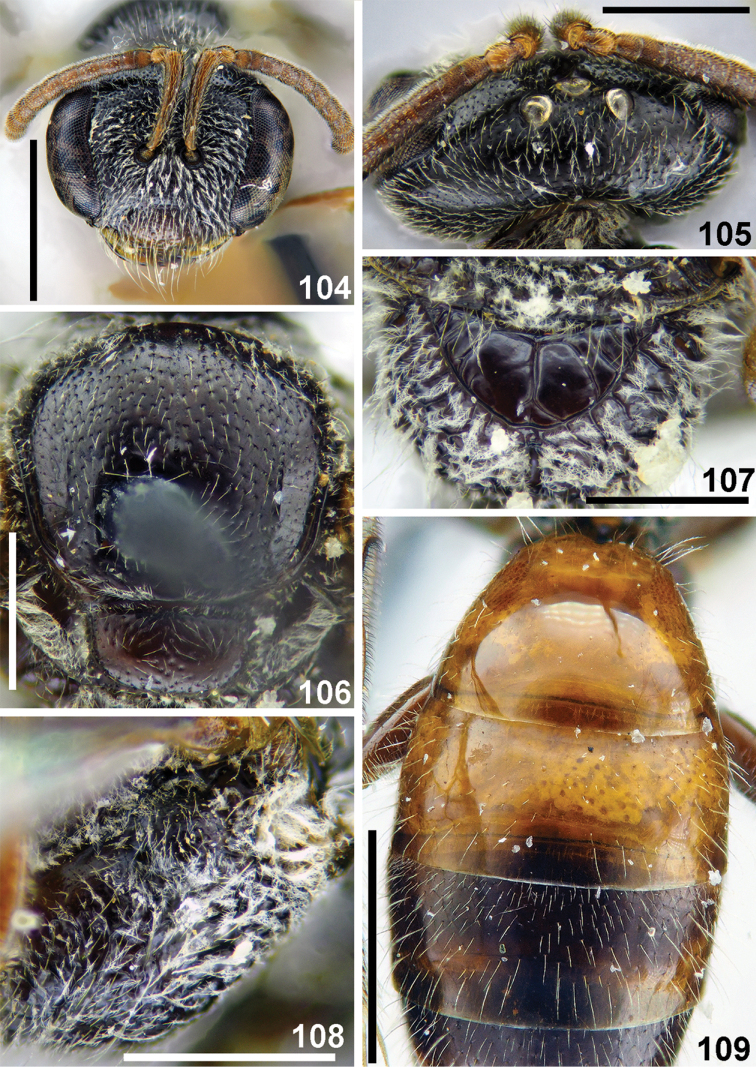
*Sphecodes pseudoredivivus* Astafurova & Proshchalykin, sp. nov., female, holotype **104** head, frontal view **105** vertex, dorsal view **106** scutum, dorsal view **107** propodeum, dorsal view **108** hypoepimeral area and mesepisternum, lateral view **109** T1–T3, dorsal view. Scale bars: 1.0 mm (**104, 109**), 0.5 mm (**105–108**).

Figures 103 , 104–109

##### Type material.

***Holotype***: ♀, Laos, Louang Prabang prov., 20°33'N, 102°14'E, Ban Songcha, 1200 m, 24.IV–16.V.1999 (OLBL/PCMS), Fig. 103.

##### Diagnosis.

This species is sculpturally closest to *Sphecodes malayensis* Blüthgen, 1927, *S. redivivus* Blüthgen, 1927 and *S. sauteri* Meyer, 1925 (refer to diagnosis of *S. sauteri* below) and possibly is the unknown female of *S. redivivus* owing to a similar sculpture of the hypoepimeral area.

##### Description.

**Female** (holotype, Fig. 103). Total body length 5.0 mm, fore wing 4.4 mm. Head (Fig. 104) black (except reddish antenna, yellow mouthparts and lower clypeus); transverse, ca. 1.2 times as wide as long; preoccipital carina absent; vertex weakly elevated, distance from top of head to upper margin of a lateral ocellus at most a half ocellar diameter as seen in frontal view and ca. 2 diameters as seen in dorsal view; mandibles simple; labrum short, semi-oval, 0.2 times as long as basalwidth; F1 transverse, 0.7 times as long as wide; F2 square; F3 1.2 times as long as wide; face densely punctate (15–20 μm / 0.5–1.5); ocello-ocular area (Fig. 105) and gena shiny, sparsely punctate (ca. 10 μm / 1–3); paraocular and supraclypeal areas with relatively dense plumose setae, but not obscuring integument; gena with sparse thin pubescence.

Mesosoma black with legs yellow-brownish to light yellow; wings hyaline, with weak yellowish darkening and light brown stigma and veins; stigma wide, 0.4 times as long as wide; hind wing with angle between basal (M) and cubital (Cu) veins ca. 90°, costal margin with five hamuli; mesoscutum and mesoscutellum (Fig. 106) finely punctate (10–20 μm / 1–4); metafemur elongate, weakly enlarged in the proximal half, maximum width 0.3 times its length; hypoepimeral area smooth with coarse and dense punctures (Fig. 108), mesepisternum areolate-punctate to rugose, but smooth with minute punctures along posterior margin. Propodeal triangle (metapostnotum) with a few coarse longitudinal wrinkles and shining smooth large interspaces (Fig. 107); lateral and vertical parts of propodeum roughly rugose with dense short plumose setae almost obscuring integument.

Metasomal T1 almost impunctate with a few minute setae pores (Fig. 109); remaining terga with sparse minute setae pores; marginal zones impunctate; T1–T2 red, remaining terga red-brownish; pygidial plate shining and very narrow, 0.4 times as wide as metabasitarsus. Sterna finely tessellate with dense shallow setae pores.

**Male** unknown.

##### Etymology.

The species name highlights the morphological similarity with *S. redivivus* Blüthgen.

##### Distribution.

Only known from the type locality in Laos.

#### Sphecodes samarensis

Taxon classificationAnimaliaHymenopteraHalictidae

Blüthgen, 1927

E8149489-1A43-57AC-A740-F09481830944

Figures 110–114

Sphecodes samarensis Blüthgen, 1927: 73, Fig. 19a–e, ♂ (holotype: ♂, Philippines, Insel Samar, Baker leg.; ZMHB, examined, illustrated in Fig. 125).

##### Diagnosis.

This species is closest to *Sphecodes bakeri* (refer to diagnosis of *S. bakeri*, above). The male is also similar to *S. biroi* Friese, 1909 and *S. laticeps* Meyer, 1920 owing to a similar structure, sculpture and coloration of the body, including the shape of the male genitalia. The species differs from *S. laticeps* by the areolate and less elevated vertex with distance from top of head to upper margin of lateral ocellus half or one of a lateral ocellar diameter as seen in frontal view (versus shining vertex with interspaces between punctures and distance from top of head to upper margin of lateral ocellus one and a half or two lateral ocellar diameters. The female of *S. samarensis* is unknown, but these features would work in both sexes). From *S. biroi* the species differs in the shape of tyloids with a glabrous medial spot on ventral surface of flagellomeres (versus tyloids usually covering entire ventral flagellar surface or sometimes with a small non-setae spot on basal flagellomeres). The unknown female is probably closest to *S. duplex* and *S. bakeri*.

##### Descriptive notes.

**Figures 110–114.**
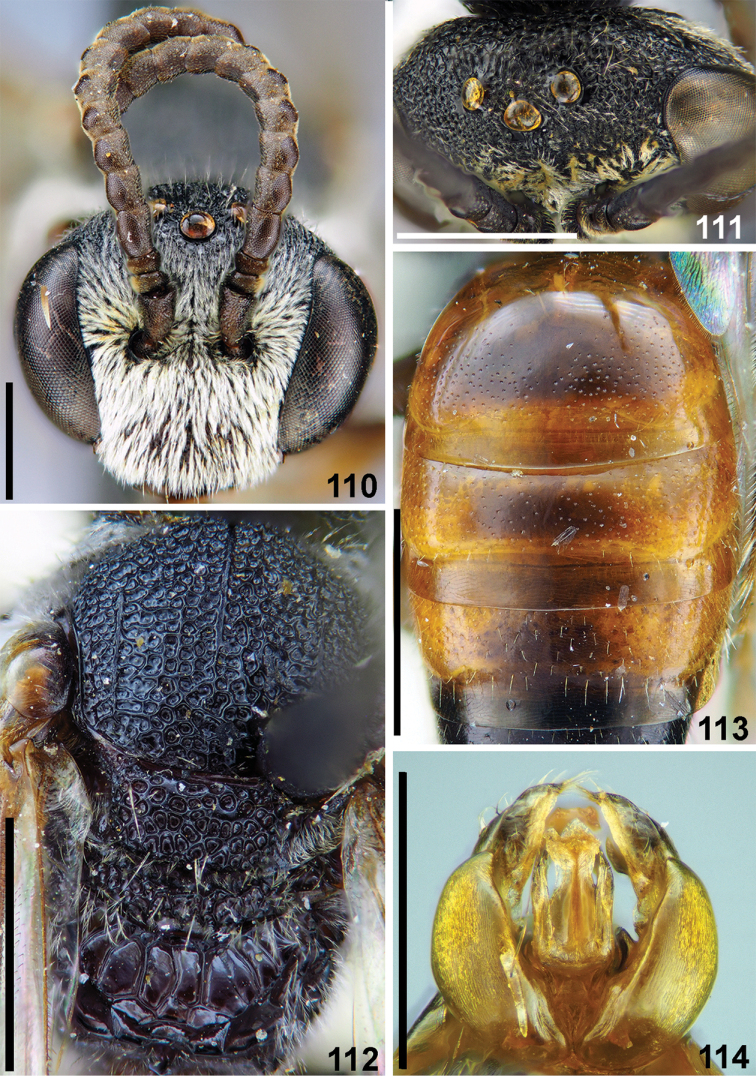
*Sphecodes samarensis* Blüthgen, male **110** head, frontal view **111** vertex, dorsal view **112** mesosoma, dorsal view **113** T1–T3, dorsal view **114** genitalia, dorsal view. Scale bars: 1.0 mm.

Wings with weak yellow-brownish darkening; hind wing with the angle between basal (M) and cubital (Cu) veins almost 80°, costal margin with seven hamuli. Lateral preoccipital carina present. **Male.** Total body length 5–6.5 mm. Head (Fig. 110) transverse, ca. 1.25 times as wide as long; vertex weakly elevated with the distance from top of head to upper margin of a lateral ocellus half or one of a lateral ocellar diameter as seen in frontal view; antennae (Fig. 110) short, reaching posterior margin of mesoscutum, F2 ca. 1.5 times as long as wide, remaining flagellomeres ca. 1.2 times as long as wide; tyloids well developed, covering entire lateral flagellar surfaces and peripheral part of ventral surface (ventral surface medially with glabrous round spot); face and vertex (Fig. 111) finely areolate-punctate; face (below and above the antennal sockets) with adpressed white pubescence obscuring integument, gena with sparser pubescence. Mesoscutum and mesoscutellum areolate (Fig. 112); propodeal triangle sculpture roughly reticulate-rugose, forming a row of large deep longitudinal cells; lateral parts of propodeum rugose with large smooth shiny interspaces. Metasomal terga (Fig. 113) with fine punctures (10–15 μm /1–3); marginal zones impunctate; T1–T3 red; gonocoxite dorsally without impression; gonostylus as on Fig. 114. **Female** unknown.

##### Material examined.

Indonesia: 1 ♂, Sumatra, 500 m, Sirggalang Annai Valley n.r., 14.X.2005, S. Jakl (OLBL/PCMS); Malaysia: 1 ♂, Pahang, 30 km NE Raub, 300 m, Lala Lembik, 3°56'N, 101°38'E, IV-V.2002, E. Jendek, O. Sausa (OLBL/PCMS); Philippines: 4 ♂♂ (holotype and paratypes), Insel Samar, Baker [leg.] (ZMHB).

##### Published records.

Blüthgen 1927: 73 (Philippines), Ascher and Pickering 2019 (Philippines).

##### Distribution.

*Indonesia, *Malaysia, Philippines.

#### Sphecodes sauteri

Taxon classificationAnimaliaHymenopteraHalictidae

Meyer, 1925

E517FFE2-9857-55D5-A41F-7CA1209E41F5

Figures 115–119 , 120–126

Sphecodes sauteri Meyer, 1925: 10, ♂ (holotype: ♂, “Formosa [Taiwan], Mt. Hoozan, 1910, III, Sauter [leg.]”; HNHM, examined, Fig. 125).

##### Diagnosis.

This species is sculpturally closest to *Sphecodes malayensis* Blüthgen, 1927, *S. pseudoredivivus* sp. nov. and *S. redivivus* Blüthgen, 1927 including a scarcely punctate metasomal terga and smoothed hypoepimeral area (differences between males of these species are outlined in Table 3). These species belong to the same species-group and females of *S. sauteri* and *S. pseudoredivivus* have simple mandibles, and the unknown females of *S. malayensis* and *S. redivivus* probably have simple mandibles as well. The female of *S. sauteri* differs from *S. pseudoredivivus* in having shorter flagellomeres from F3 onward (ca. 0.9–1.0 versus 1.2) and a scarcely punctate hypoepimeral area (versus dense punctures separated by approximately a puncture diameter).

**Table 3.** Differences between males of *Sphecodes sauteri*, *S. malayensis*, and *S. redivivus*.

Characters	*Sphecodes* species		
	* sauteri *	* malayensis *	* redivivus *
Head	1.2 times as wide as long	1.25 times as wide as long	1.2 times as wide as long
Tyloids	Semi-oval across 1/3 basal flagellar surfaces	Semi-oval across 1/2 basal flagellar surfaces	Semi-oval across 1/4 basal flagellar surfaces
F3	1.2 as long as wide	Square	Square
Hypoepimeral area	Smooth with tiny and sparse punctures	Smooth with a few microscopical punctures	Smooth to finely rugulose with dense punctures
Number of hamuli	6-7	5	5
Antennae coloration	Red	Brown	Brown

##### Descriptive notes.

**Figures 115–119.**
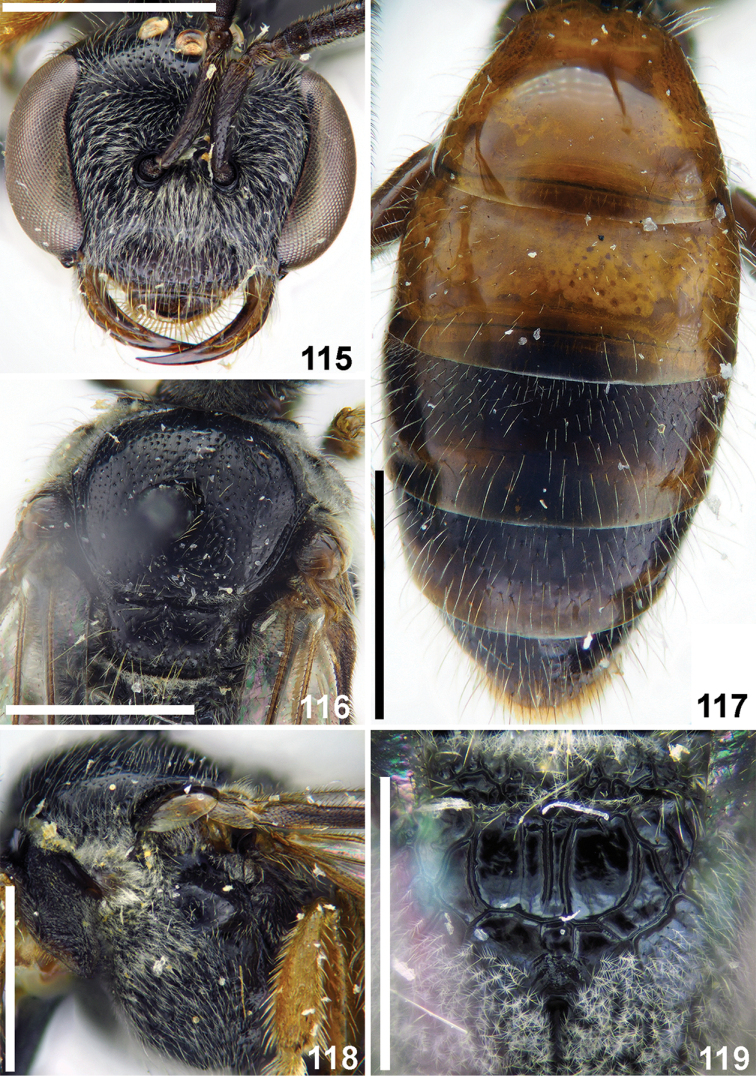
*Sphecodes sauteri* Meyer, female **115** head, frontal view **116, 118** mesosoma, dorsal view (**116**), lateral view (**118**) **117** metasoma, dorsal view **119** propodeum, dorsal view. Scale bars: 1.0 mm.

**Figures 120–126.**
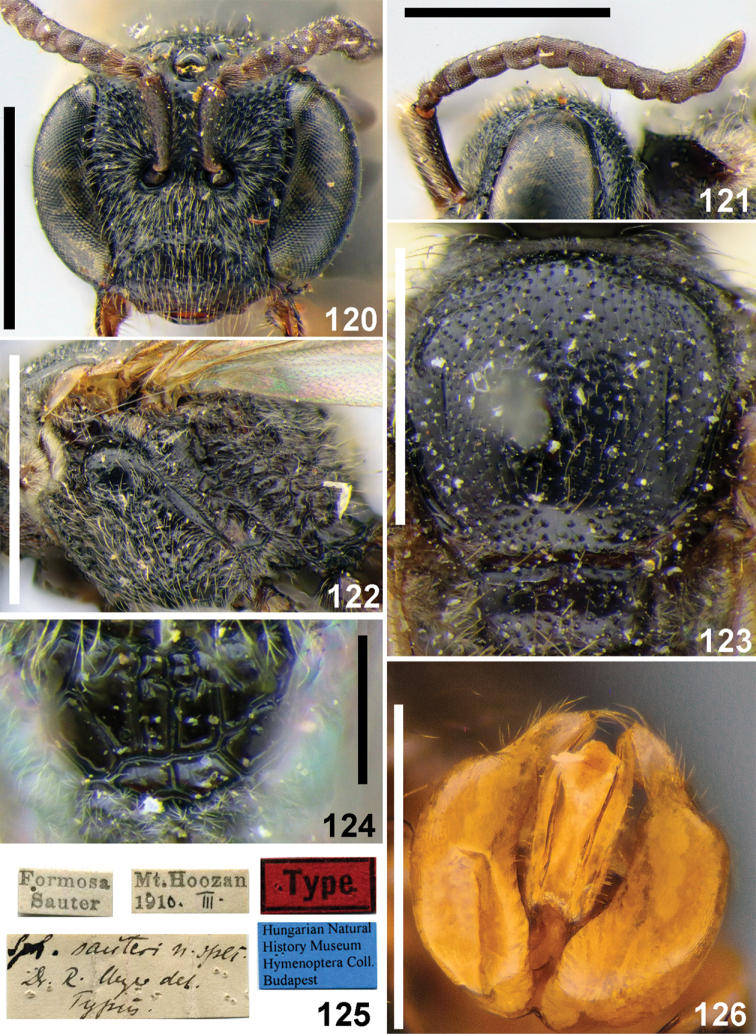
*Sphecodes sauteri* Meyer, male, holotype (**120–125**) **120** head, frontal view **121** antennae, lateral view **122** mesepisternum, lateral view **123** scutum, dorsal view **124** propodeum, dorsal view **125** holotype labels **126** genitalia, dorsal view. Scale bars: 1.0 mm (**120–123**), 0.5 mm (**124, 126**).

Wings with weak yellow-brownish darkening; hind wing with the angle between basal (M) and cubital (Cu) veins ca. 90°, costal margin with six or seven hamuli. Preoccipital carina absent. **Female** (new). Total body length 6 mm. Head (Fig. 115) strongly transverse, ca. 1.2 times as wide as long; vertex not elevated as seen in frontal view; mandible simple; labrum short, semi-oval, 0.2 times as long as basal width; F2 and F3 nearly square, ca. 0.9 times as long as wide; ocello-ocular area shining, sparsely punctate (ca. 15 μm / 0.5–3). Gena smooth and shining, with sparse setae pores; paraocular and supraclypeal areas with relatively dense plumose setae, but not obscuring integument. Gena with sparse pubescence. Mesosoma (Figs 116, 118, 119) and metasoma (Fig. 117) sculptured as in the male; lateral and vertical parts of propodeum with dense short plumose setae, obscuring integument. Metasoma red, pygidial plate as wide as metabasitarsus. **Male.** Total body length 5.0–5.5 mm. Head (Fig. 120) transverse, ca. 1.2 times as wide as long; vertex not elevated as seen in frontal view; antennae short (Fig. 121), reaching middle of mesoscutum, flagellomeres (from F2 onward) ca. 1.2 times as long as wide; tyloids semi-oval across basal 1/4–1/3 of flagellar surfaces; face and ocello-ocular area with punctures (20–25 μm) separated by 0.5–2puncture diameters; face and gena with sparse pubescence. Mesoscutum and mesoscutellum (Fig. 123) densely and finely punctate (20–25 μm / 0.5–2); hypoepimeral area smooth with minute and sparse punctures (Fig. 122); propodeal triangle coarsely reticulate-rugose with shining large interspaces between wrinkles (Fig. 124); lateral and vertical parts of propodeum with dense short plumose setae, almost obscuring integument. Metasomal T1 almost impunctate; remaining terga with sparse setae pores; T1–T3 red or brownish; gonocoxite dorsally without impression; gonostylus short, as in Fig. 126.

##### Material examined.

Laos: 1 ♀, Phongsaly prov., Phongsaly env., 21°41'N, 102°06'E, 1500 m, 28.V.-20.VI.2003, V. Kuban (OLBL/PCMS).

##### Distribution.

*Laos, China (Taiwan).

#### Sphecodes sikkimensis

Taxon classificationAnimaliaHymenopteraHalictidae

Blüthgen, 1927

44A46265-C8F2-5C14-A575-3287F3A20882

Sphecodes sikkimensis Blüthgen, 1927: 54, Fig. 12a, ♀ (syntypes: ♀♀, Sikhim [India], 6.97., Rungit Tal, 1000', Coll. Bingham; ZMHB, examined).

##### Diagnosis.

This species differs noticeably from other described Oriental species with a lateral preoccipital carina by a combination of large total body length (12–15 mm) and the greatest number (12–15) of hamuli (other large oriental species are usually at most 12 mm in length and have hind wings with at most eleven or twelve hamuli).

##### Descriptive notes.

Wings with strong brownish darkening and metallic violet lustre; hind wing with the angle between basal (M) and cubital (Cu) veins ca. 80°, costal margin with 12–15 hamuli. **Female.** Total body length 12–15 mm. Head transverse, 1.25 times as wide as long; vertex elevated with the distance from top of head to upper margin of a lateral ocellus ca. two lateral ocellar diameters as seen in frontal view; labrum short, semi-oval, 0.45 times as long as basal width; face and ocello-ocular area areolate-punctate; paraocular areas and gena with sparse pubescence not obscuring integument. Mesoscutum and mesoscutellum areolate-punctate (50–100 μm). Propodeal triangle (metapostnotum) coarsely reticulate-rugose; mesepisternum reticulate-rugose. Metasoma red, coarsely and densely punctate (ca. 25 μm), sparser on T1. Marginal zone impunctate, except T1 with fine punctures (ca. 10 μm); pygidial plate dull, 1.2 times as wide as metabasitarsus. **Male** unknown.

##### Material examined.

Laos: 1 ♀, Prov. Hua Phan, Phou Pan, Umg. Ort Ban Saleui, 20°13'N, 103°59'E, 1350–1900 m, 28.IV.2012, C. Holzschuh (OLBL/PCMS); Myanmar: 1 ♀, Nam Tamai, 3000 ft, 9.I.1931, F. Kingdon Ward (NHMUK 013380357).

##### Distribution.

*Laos, *Myanmar, NE India, China (Guangdong).

#### Sphecodes simlaensis

Taxon classificationAnimaliaHymenopteraHalictidae

Blüthgen, 1924

33D97A39-CB0A-5EBD-817C-18EA3B889247

Figures 127–129 , 130–134

Sphecodes simlaensis Blüthgen, 1924: 514–515, ♀ (syntypes: 2 ♀♀, India, Simla, VIII. and IX.[18]98, Nurse leg.; were not found in NHMUK).
Sphecodes simlaellus Blüthgen, 1927: 46–48, Fig. 8, ♂ (lectotype (**designated here**): ♂, Simla [India, Himachal Pradesh], 8.98 // Col. C.G. Nurse Collection. 1920-72 // Sph. simlaensis n. sp., ♂, P. Blüthgen det. // Type; ZMHB, examined; paralectotype: 1 ♂ [without head]: Type // Simla, Nurse 9. 98 // Col. C.G. Nurse Collection. 1920-72 // Sph. simlaensis, Type P. Blüthgen det. // B.M.Type HYM.17a548 // NHMUK 0133803332; examined). Syn. nov.

##### Diagnosis.

This species is close to the Palaearctic *Sphecodes geoffrellus* (Kirby 1802) owing to a similar structure, sculpture, coloration of the body and shape of the male gonostylus. Females of *S. simlaensis* and *S. geoffrellus* are difficult to distinguish morphologically, but the male of *S. simlaensis* is easy discerned by the weakly developed tyloids, covering at most 1/4 of the basal ventral surfaces of the flagellomeres, Fig. 131 (versus at least 4/5 in *S. geoffrellus*). Structurally, the male of *S. simlaensis* is also close to *S. shillongensis* Blüthgen, 1927, but differs in the shape of the gonostylus which has a membranous part (lacking in *S. shillongensis*).

##### Descriptive notes.

**Figures 127–129.**
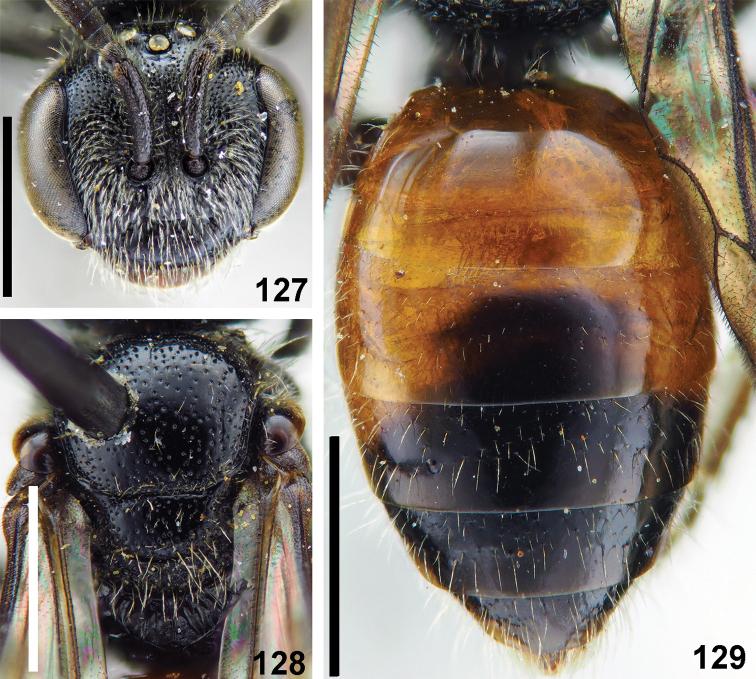
*Sphecodes simlaensis* Blüthgen, female **127** head, frontal view **128** mesosoma, dorsal view **129** metasoma, dorsal view. Scale bars: 1.0 mm.

**Figures 130–134.**
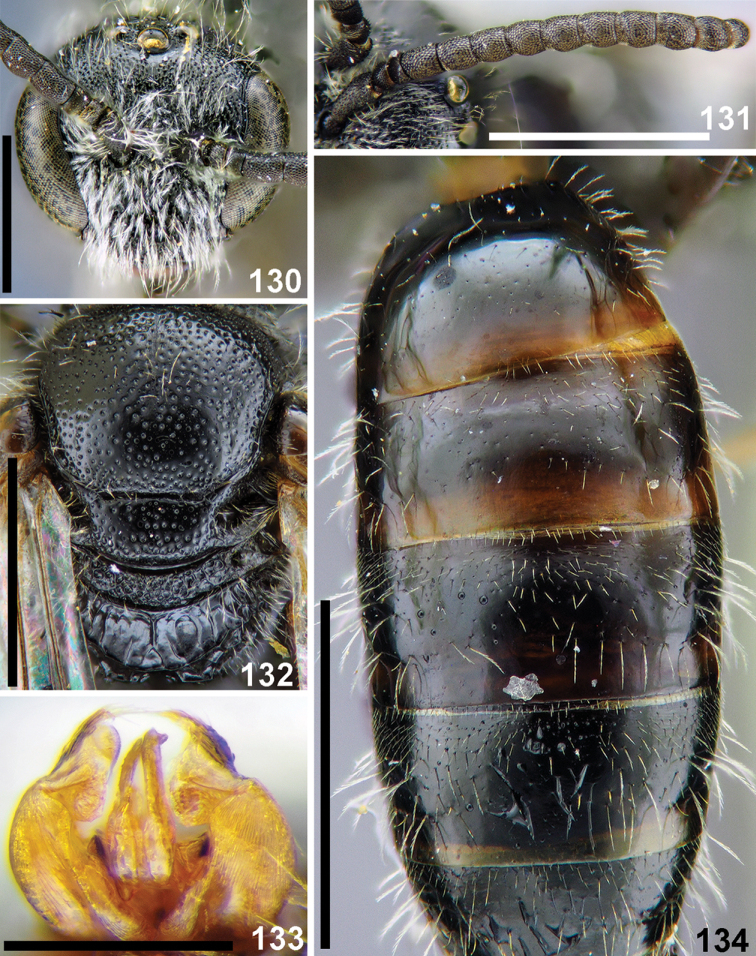
*Sphecodes simlaensis* Blüthgen, male **130** head, frontal view **131** antennae, ventral view **132** mesosoma, dorsal view **133** genitalia, dorsal view **134** metasoma, dorsal view. Scale bars: 1.0 mm (**130–132, 134**), 0.5 mm (**133**).

Wings with weak yellowish or brownish darkening; hind wing with basal vein strongly curved with angle between basal (M) and cubital (Cu) veins ca. 80°, costal margin with five hamuli. Preoccipital carina absent. **Female.** Total bodylength 5–5.5 mm. Head (Fig. 127) weakly transverse, at most 1.2 times as wide as long; vertex not elevated as seen in frontal view; F1 and F2 transverse, 0.6–0.8 times as long as wide, F3 almost square, 0.9 times as long as wide; clypeus with punctures separated by 0.5–2 puncture diameters; ocello-ocular area with fine punctures separated by 1–3 puncture diameters; face and gena with sparse pubescence. Mesoscutum and mesoscutellum (Fig. 128) with punctures (15–20 μm) separated by 1–4 puncture diameters; hypoepimeral area coarsely reticulate. Propodeal triangle (metapostnotum) with coarse longitudinal wrinkles and shiny interspaces. Metasomal T1 impunctate, remaining terga basally with fine sparse setae pores (Fig. 129); marginal zones impunctate; T1–T3 red, pygidial plate 0.7 times as wide as metabasitarsus. **Male.** Total body length 5–5.5 mm. Head (Fig. 130) slightly transverse, 1.1 times as wide as long; vertex not elevated as seen in frontal view; antenna reaching posterior margin of mesoscutum; F2 1.4 times as long as wide, remaining flagellomeres almost square, ca. 1.1 times as long as wide, tyloids weakly developed, semi-oval across at most basal 1/4 of flagellar ventral surfaces (Fig. 131); ocello-ocular area shining, with fine punctures separated by 1–3 puncture diameters; face with pubescence obscuring integument below antennal stockers and sparser above. Mesoscutum medially with punctures (15–25 μm) separated by 0.5–3 puncture diameters, becoming denser peripherally (Fig. 132). Propodeal and metasomal sculpture as in the female; terga brownish (Fig. 134); gonocoxite dorsally with impression; gonostylus with small rectangular membranous part (Fig. 133).

##### Material examined.

Laos: 1 ♂, Phongsaly pr., Phogsaly env., 1500 m, 21°41'N, 102°06'E, VII.2003, Pacholatko (OLBL/PCMS); 1 ♂, idem, 6–17.V.2004, V. Kuban (OLBL/PCMS).

##### Distribution.

*Laos, India (Himachal Pradesh), Pakistan.

#### Sphecodes turneri

Taxon classificationAnimaliaHymenopteraHalictidae

Cockerell, 1916

2AB22CD7-37C6-5E0C-9DCD-3EA4C3AC9A5A

Figures 135–138 , 139–144

Sphecodes turneri Cockerell, 1916: 430, ♀ (holotype: ♀, India, Assam, Shilong, 5.03., B. Turner, 1905-125. 17a.561; NHMUK 013380320; examined).

##### Diagnosis.

This species differs from other species of the genus by having forewings with two submarginal cells (Fig. 143) (versus three cells in other *Sphecodes* species).

##### Descriptive notes.

**Figures 135–138.**
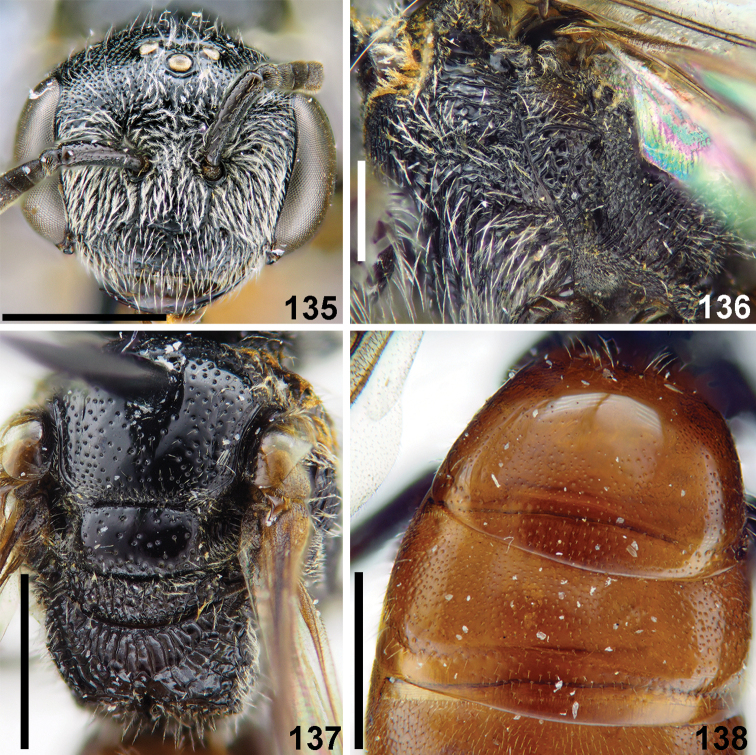
*Sphecodes turneri* Cockerell, female **135** head, frontal view **136** mesepisternum, lateral view **137** mesosoma, dorsal view **138** T1-T2, dorsal view. Scale bars: 1.0 mm (**135, 137, 138**), 0.5 mm (**136**).

**Figures 139–144.**
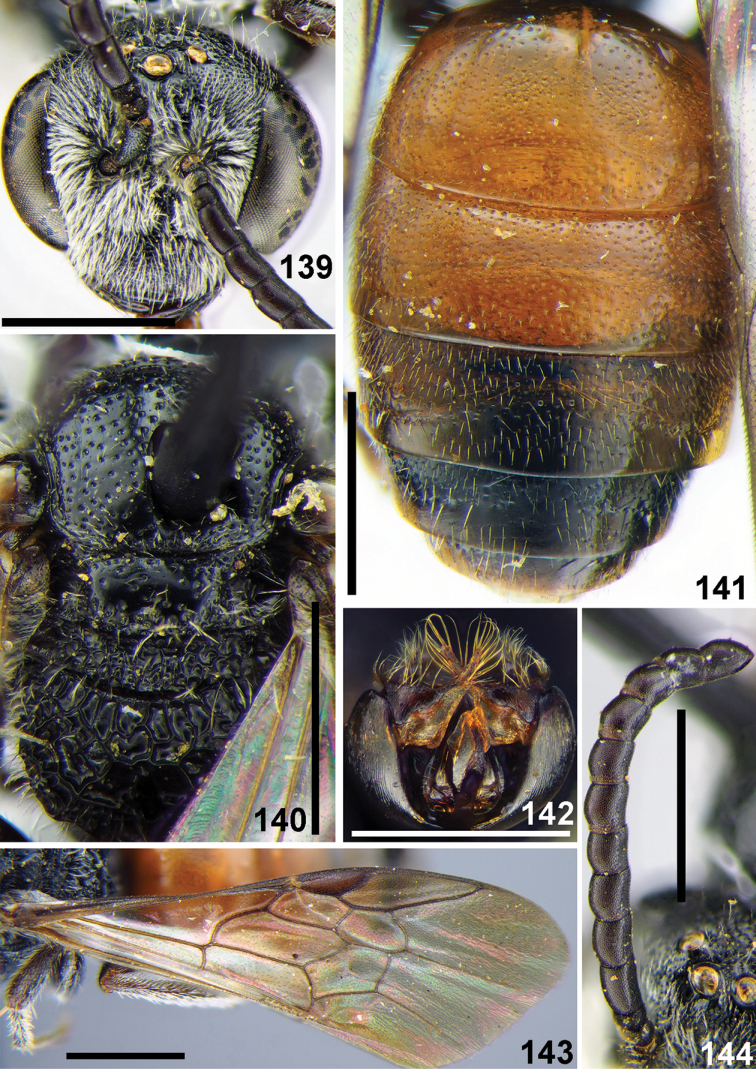
*Sphecodes turneri* Cockerell, male **139** head, frontal view **140** mesosoma, dorsal view **141** metasoma, dorsal view **142** genitalia, dorsal view **143** forewing, lateral view **144** antennae, ventro-lateral view. Scale bars: 1.0 mm (**139–141, 143, 144**), 0.5 mm (**142**).

Wings with brownish darkening; hind wing with the angle between basal (M) and cubital (Cu) veins ca. 70°, costal margin with eight or nine hamuli. **Female.** Total body length 8–9 mm. Head transverse (Fig. 135), ca. 1.3 times as wide as long; vertex elevated with distance from top of head to upper margin of lateral ocellus ca. one and a half lateral ocellar diameter as seen in frontal view; ocello-ocular area shining, with punctures separated by 0.5–2 puncture diameters; paraocular and supraclypeal areas with dense adpressed white plumose pubescence, gena with sparser pubescence. Mesoscutum (Fig. 137) irregularly punctate, denser peripherally and with large interspaces medially (20–35 μm / 0.5–5); mesoscutellum sparsely punctate withlarge impunctate interspaces; propodeal triangle (metapostnotum) with longitudinal parallel wrinkles (Fig. 137); lateral parts of propodeum striate-rugose; mesepisternum reticulate-rugose (Fig. 136). Metasoma with a mixture of minute and coarse punctures (5–25 μm / 1–3) (Fig. 138); marginal zones T1 entirely and T2 medially punctate Pygidial plate narrow, 0.4 times as wide as metabasitarsus; T1–T4 red. **Male** (new). Total body length 7.0–8.5 mm. Head (Fig. 139) weakly transverse, 1.15 times as wide as long; vertex elevated, with distance from top of head to upper margin of lateral ocellus ca. one and a half of a lateral ocellar diameter as seen in frontal view; antenna long, reaching mesoscutellum, F2 1.7 times as long as wide, remaining flagellomeres ca. 1.2 times as long as wide; tyloids weakly developed, narrowly semicircular across atmost 1/4 of the basal flagellar surfaces (Fig. 144). Face and ocello-ocular area densely punctate, the punctures separated by at most 0.5 of a puncture diameter. Mesoscutum coarsely punctate (20–30 μm / 0.5–3); mesoscutellum irregularly punctate with large interspaces (Fig. 140). Propodeal triangle coarsely reticulate-rugose (Fig. 140); mesepisternum reticulate-rugose. Metasoma (Fig. 141) with a mixture of minute and coarse punctures, 5–20 μm); marginal zones of T1 and T2 punctate; gonocoxite dorsally without impression; gonostylus with a long and narrow apical process as in Fig. 142.

##### Material examined.

Laos: 1 ♀, 1 ♂, Prov. Hua Phan, Phou Pan, Umg. Ort Ban Saleui, 20°13'N, 103°59'E, 1350–1900 m, 15.IV.2012, C. Holzschuh & locals (OLBL/PCMS).

##### Distribution.

*Laos, India (Meghalaya).

##### Remarks.

It is noteworthy that the other cleptoparasitic genus *Nomada* Scopoli (Apidae) also has a small group of species with two submarginal cells (Proshchalykin and Lelej 2010).

## Discussion

The most important figures, on which the study is based, are shown in Table 1, which assigns the individual species to the countries of Southeast Asia, with the respective totals and number of collection points. Amongst all bees in the collections we studied, the proportion of specimens from the Oriental Region belonging to the genus *Sphecodes* present turned out to be scanty, which suggests that the genus in this region is extremely rare. Although the number of species recorded here (approximately 50) is less than in the Palaearctic Region (approximately 70), this number will probably exceed the number of Palaearctic species eventually as further new species are described. In total, 31 species of *Sphecodes* are recorded from Southeast Asia (Table 1) and only eleven of these have a distribution beyond the studied region (India, Pakistan, China). However, the record of 20 other species confined to Southeast Asia does not indicate a large degree of endemicity of the fauna, but rather suggests an incomplete knowledge of the distribution of the oriental fauna of *Sphecodes*.

Most species recorded in Southeast Asia have montane distributions and are found up to 1900 m. The range of *S. biroi*, which is distributed from New Guinea to India, is the widest among Southeast Asian species. *Sphecodes simlaensis*, *S. montanus*, *S. sikkimensis*, and *S. fumipennis* are also probably widespread in the mountainous areas of the Oriental Region.

Morphologically, a large proportion of Southeast Asian species have close affinities to some of the Palaearctic species or belong to one of the Palaearctic species groups. *Sphecodes engeli* belongs to the *hyalinatus* species group (for the composition of Palaearctic species groups see Astafurova and Proshchalykin 2017a); *S. discoverlifei* is similar to *S. crassus* Thomson, 1870; *S. montanus* resembles the Eastern Palaearctic *S. kozlovi* Astafurova & Proshchalykin, 2015 and *S. simillimus* Smith, 1873; *S. sauteri*, *S. malayensis*, *S. pseudoredivivus* and *S. redivivus* are the closest to several small Palaearctic species with simple mandibles which lack an inner tooth (i.e., *S. armeniacus* Warncke,1992, *S. longuloides* Blüthgen, 1923, *S. hirtellus* Blüthgen, 1923, *S. longulus* Hagens, 1882, *S. puncticeps* Thomson, 1870, *S. turanicus* Astafurova & Proshchalykin, 2017, and *S. trjapitzini* Astafurova & Proshchalykin, 2018); *S. simlaensis* is similar to the Palaearctic *S. geoffrellus* (Kirby 1802). Widespread Palaearctic *S. scabricollis* Wesmael, 1835 is similar to a significant number of Southeast Asian species (*S. bakeri*, *S. binghami*, *S. biroi*, *S. distinctus*, *S. duplex*, *S. formosanus*, *S. howardi*, *S. insularis*, *S. kershawi*, *S. laticeps*, *S. samarensis*, *S. sibuyanensis*, *S. sikkimensis*, *S. takaensis*, *S. tristellus*, *S. rotundiceps*, and *S. ilyadadaria*) by the presence of a lateral preoccipital carina and densely punctate mesosoma. At the same time two species have a unique combination of characters that has no analogue to any of the known Palaearctic and Oriental species: *S. turneri* with two submarginal cells in the forewing and *S. brunneipes* with a combination of simple mandibles in the female and a lateral preoccipital carina.

It is quite certain that new species will be found in further studies, and through synonymy and the association of sexes described as separate species, numerous changes in the species spectrum can be expected in the future.

## Supplementary Material

XML Treatment for Sphecodes bakeri

XML Treatment for Sphecodes binghami

XML Treatment for Sphecodes biroi

XML Treatment for Sphecodes brunneipes

XML Treatment for Sphecodes chaprensis

XML Treatment for Sphecodes discoverlifei

XML Treatment for Sphecodes distinctus

XML Treatment for Sphecodes duplex

XML Treatment for Sphecodes engeli

XML Treatment for Sphecodes fumipennis

XML Treatment for Sphecodes howardi

XML Treatment for Sphecodes ilyadadaria

XML Treatment for Sphecodes kershawi

XML Treatment for Sphecodes laticeps

XML Treatment for Sphecodes montanus

XML Treatment for Sphecodes pseudoredivivus

XML Treatment for Sphecodes samarensis

XML Treatment for Sphecodes sauteri

XML Treatment for Sphecodes sikkimensis

XML Treatment for Sphecodes simlaensis

XML Treatment for Sphecodes turneri
